# Integrated multi-omics analyses revealed the association between rheumatoid arthritis and colorectal cancer: MYO9A as a shared gene signature and an immune-related therapeutic target

**DOI:** 10.1186/s12885-024-12466-5

**Published:** 2024-06-10

**Authors:** Zhi-Qing Zhan, Ze-Min Huang, Qi-Wen Lan, Yu-Hua Luo, Jia-Xin Li, Ya-Fang Zheng, Ying-Zhou Chen, Pei-Zhen Chen, Tian-Ye Luo, Baoqing Sun, Zhangkai J Cheng

**Affiliations:** 1 Department of Clinical Laboratory, National Center for Respiratory Medicine, National Clinical Research Center for Respiratory Disease, State Key Laboratory of Respiratory Disease, Guangzhou Institute of Respiratory Health, The First Affiliated Hospital of Guangzhou Medical University, Guangzhou Medical University, Guangzhou, China; 2 Division of Gastroenterology and Hepatology, NHC Key Laboratory of Digestive Diseases, State Key Laboratory for Oncogenes and Related Genes, Renji Hospital, School of Medicine, Shanghai Institute of Digestive Disease, Shanghai Jiao Tong University, Shanghai, China; 3 Department of Clinical Medicine, Guangzhou Medical University, Guangzhou, China; 4 Department of Gastroenterology, West China Hospital, Sichuan University, Chengdu, Sichuan China

**Keywords:** Multi-omics analysis, Rheumatoid arthritis, Colorectal cancer, Immunotherapy, MYO9A

## Abstract

**Background:**

Our study aims to explore the relationship, shared gene signature, and the underlying mechanisms that connect rheumatoid arthritis (RA) to colorectal cancer (CRC).

**Methods:**

Mendelian randomization (MR) analysis was conducted to assess the causality between RA and CRC. Summary statistic data-based Mendelian randomization (SMR) leveraging eQTL data was employed to identify the CRC-related causal genes. Integrated analyses of single-cell RNA sequencing and bulk RNA sequencing were employed to comprehensively investigate the shared gene signature and potential mechanisms underlying the pathogenesis of both RA and CRC. Predictive analysis of the shared hub gene in CRC immunotherapy response was performed. Pan-cancer analyses were conducted to explore the potential role of MYO9A in 33 types of human tumors.

**Results:**

MR analysis suggested that RA might be associated with a slight increased risk of CRC (Odds Ratio = 1.04, 95% Confidence Interval = 1.01–1.07, *P* = 0.005). SMR analysis combining transcriptome analyses identified MYO9A as a causal gene in CRC and a shared gene signature in both RA and CRC. MYO9A may contribute to tumor suppression, while downregulation of MYO9A may impact CRC tumorigenesis by disrupting epithelial polarity and architecture, resulting in a worse prognosis in CRC. Additionally, MYO9A shows promise as a powerful predictive biomarker for cancer prognosis and immunotherapy response in CRC. Pan-cancer analyses demonstrated MYO9A may have a protective role in the occurrence and progression of various human cancers.

**Conclusion:**

RA might be associated with a slight increased risk of CRC. MYO9A is a shared gene signature and a potential immune-related therapeutic target for both CRC and RA. Targeting the MYO9A-mediated loss of polarity and epithelial architecture could be a novel therapeutic approach for CRC.

**Supplementary Information:**

The online version contains supplementary material available at 10.1186/s12885-024-12466-5.

## Introduction

Colorectal cancer (CRC) is a prominent and pressing issue in global health, ranking as the second leading cause of cancer-related deaths and the third most prevalent cancer worldwide [1]. Rheumatoid arthritis (RA) is a prevalent autoimmune inflammatory joint disease, with a global prevalence estimated at 24.5 million individuals [2]. Recent evidence increasingly associates RA with CRC. As an immune-mediated inflammatory disease, RA exhibits heightened inflammatory responses and impaired anti-inflammatory mechanisms, potentially compromising the gut mucosa’s barrier function via the IL-23/TH17/IL-17 pathway and suppressing cytotoxic T-cell-mediated antitumor immune surveillance [3]. In addition, Flak MB et al. reported that inflammatory arthritis contributes to intestinal barrier dysfunction, which, conversely, leads to inflammation, dysbiosis, and an increased susceptibility to developing RA [4]. Emerging research has underscored the substantial contribution of the gut microbiota to the pathogenesis of both RA and CRC. For instance, Fusobacterium nucleatum, a well-recognized bacterium implicated in the initiation and progression of CRC, has also been observed to be more abundant in patients with RA and positively associated with the severity of RA [5]. Establishing the co-occurrence of CRC in individuals with RA is crucial for developing effective strategies in CRC prevention. Moreover, understanding the mechanisms behind the increased susceptibility or potential protection against CRC in RA patients could be pivotal in the discovery of innovative therapeutic interventions for both diseases. Nevertheless, prior population-based investigations exploring the association between RA and the risk of CRC have produced inconsistent findings [6, 7]. A large-scale real-world study revealed that RA patients have a 1.21-fold higher likelihood to develop any cancer compared to non-RA individuals [8]. However, this association was not detected in a longitudinal Korean population-based analysis [2]. Moreover, these studies faced methodological challenges, including medical surveillance bias and potential confounding effects of RA drug usage, which could potentially lead to misleading findings. Thus, the relationship between RA and CRC remains unclear.

**Fig. 1:**
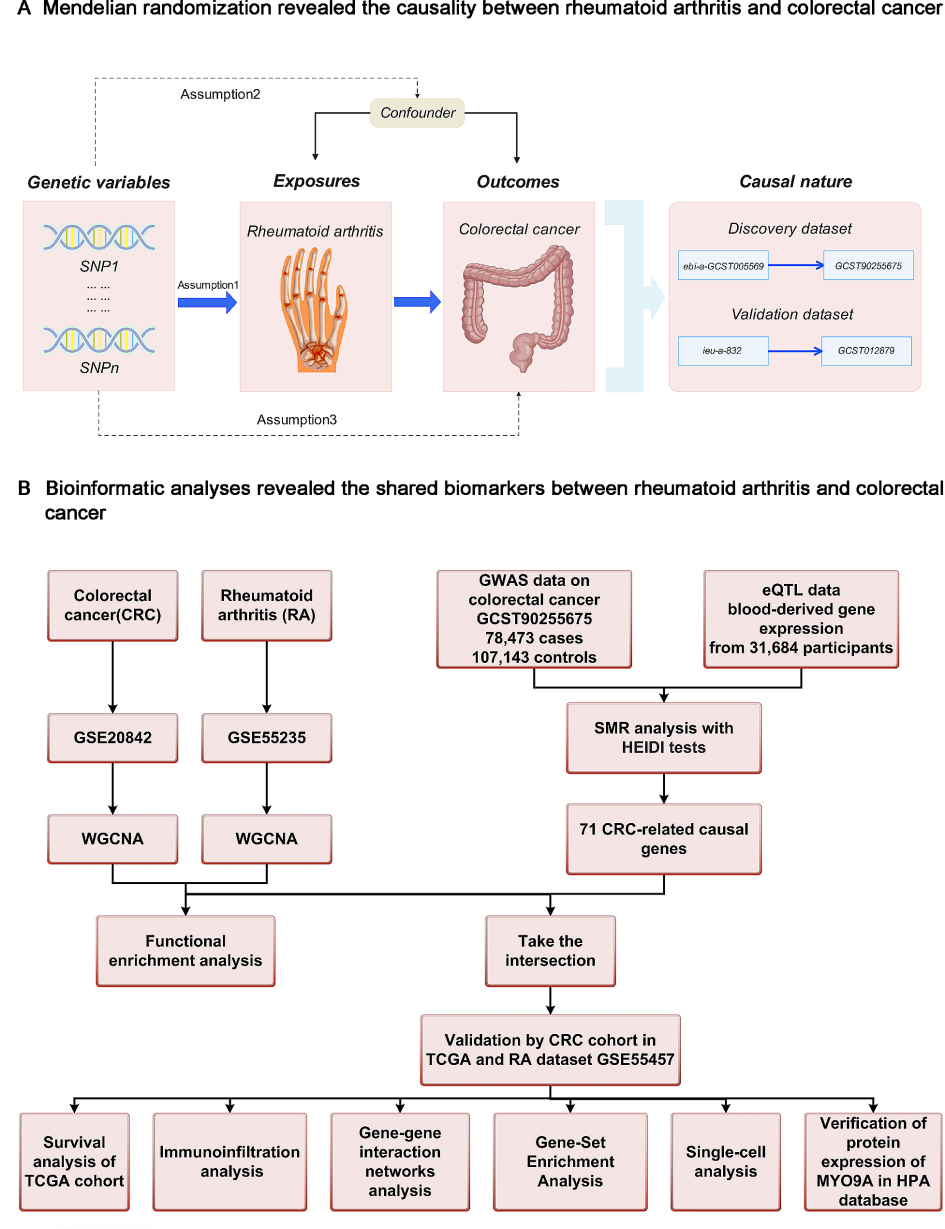
Workflow of this study design. (**A**) MR analyses. (**B**) Bioinformatic analyses

Mendelian randomization (MR) analysis employs genetic variants associated with the exposure of interest as instrumental variables (IVs) to evaluate potential causal relationships between risk factors and outcomes [9]. By utilizing genotypes that are not influenced by disease, this approach helps mitigate confounding and reverse causality biases. In this study, we aim to address the following issues: (i) Is there a causal association between RA and CRC? (ii) If so, what is the shared gene signature of both diseases? (iii) what are the potential mechanisms by which the shared gene signature mediates these two immune-related diseases, and could it serve as a common immune-related target? We performed multi-omics analyses integrating GWAS, eQTL, bulk RNA sequencing, and single-cell RNA sequencing data. The present study further expands our understanding of these two immune-mediated diseases, ranging from their co-occurring mechanisms to the predictive ability of the shared gene signature for human cancer prognosis and CRC immunotherapy response. The study design is illustrated in Fig. 1.

## Materials and methods

### MR analysis evaluating the causality between RA and CRC

#### Data source and selection of instrumental variables (IVs)

To obtain more robust results, we investigated the relationship between RA and CRC using discovery datasets and validation datasets. Genome-wide association study (GWAS) data on CRC of European ancestry were obtained from the GWAS catalog (https://www.ebi.ac.uk/gwas/). Specifically, CRC GWAS data (id: GCST90255675) consisting of 78,473 cases and 107,143 controls were used as the discovery outcome dataset [10]. CRC GWAS data (id: GCST012879) consisting of 19,948 cases and 12,124 controls were used as the validation outcome dataset [11].

To avoid bias caused by sample overlap, we used data from nonoverlapping datasets for different exposure-outcome pairs. GWAS data on RA of European ancestry were retrieved from the IEU Open GWAS Project (https://gwas.mrcieu.ac.uk/). The discovery exposure dataset was obtained from the RA GWAS data (id: ebi-a-GCST005569), which comprised 13,838 cases and 33,742 controls [12]. The validation exposure dataset was derived from the RA GWAS data (id: ieu-a-832), encompassing 14,361 cases and 43,923 controls [13]. Detailed information on the GWAS data is shown in Table S1.

IVs were selected based on the following criteria: (i) IVs should exhibit a strong association with the exposure; (ii) independence from confounders; and (iii) should not have a direct association with the outcome [9]. To meet the first assumption, we selected SNPs associated with each trait at the genome-wide significance threshold of *P* < 5 × 10^− 8^. Only SNPs with a long physical distance (≥ 10,000 kb) and less possibility of linkage disequilibrium (R^2^ < 0.001) were retained. We manually searched for pleiotropic SNPs of confounders and excluded IVs that were significantly associated (*P* < 5 × 10^− 8^) with confounders using PhenoScanner (http://phenoscanner.medschl.cam.ac.uk). Table S2 presents the SNPs that were associated with potential confounders and were excluded from the analysis. The design of this MR study is shown in Fig. 1A.

#### Statistical methods

Multiple MR approaches were employed to evaluate the relationship between RA and CRC. These methods included the inverse variance weighted (IVW) method, weighted mode, MR‒Egger, weighted median, and simple mode [9]. In addition, the presence of potential directional pleiotropy in the genetic variants was assessed using the MR‒Egger intercept’s test. To evaluate heterogeneity, Cochran’s Q test was performed [9]. The leave-one-out test is utilized to identify potential outliers and assess their possible influence [8]. The findings were considered statistically significant at *P* < 0.05. Refer to the supplementary methods for a detailed methodology.

### SMR analysis to identify CRC-related causal genes

SMR analysis has been described by Zhu et al. in detail [14]. In this study, SMR analysis was employed to identify novel causal genes related to CRC and explore their potential functional significance. CRC GWAS data (id: GCST90255675) were used for SMR analysis [10]. The eQTL data utilized in this study were sourced from the eQTLGen consortium (https://www.eqtlgen.org/phase1.html). It is widely recognized that eQTL effects observed in blood tissues can serve as a proxy for eQTL effects in the relevant tissues linked to various traits or diseases [15]. In our study, we employed summary-level data for blood-derived gene expression from a cohort of 31,684 individuals of European ancestry [16], which included 25,482 samples from whole blood and 6,202 samples from peripheral blood mononuclear cells, serving as the eQTL data for our analysis.

We utilized the heterogeneity in dependent instruments (HEIDI) test to ensure that the significant SMR results were indeed indicative of pleiotropy or causality, rather than being influenced by the less relevant linkage model [17]. The SMR and HEIDI tests were employed to investigate the enrichment of cis-eQTLs in the context of CRC. The genome-wide significance level for the SMR test was P_smr_ < 2.3 × 10^− 5^ (0.05/2131, Bonferroni correction). A *P* value threshold of P_HEIDI_ > 0.01 was considered conservative for genes exhibiting no heterogeneity. SMR analysis was implemented by the SMR software.

### Bioinformatic analyses

#### Data resources and criteria

Gene expression profiles of RA and CRC patients were obtained by searching the Gene Expression Omnibus (GEO) database (https://www.ncbi.nlm.nih.gov/geo/) using relevant keywords. Ultimately, we acquired the GSE55235 [18] and GSE20842 datasets [19] for RA and CRC, respectively. For validation purposes, we retrieved the RA dataset GSE55457 [18] from the GEO database and the CRC cohort from The Cancer Genome Atlas (TCGA) database (https://portal.gdc.cancer.gov/). The workflow is depicted in Fig. 1B. Detailed information on the datasets is shown in Table S3.

#### WGCNA analysis

WGCNA is a powerful algorithm that clusters genes, constructs modules based on shared expression patterns, and explores associations with biological traits in multiple samples [20]. Specifically, in the CRC dataset GSE20842 [19], genes in the top 65% MAD and MAD greater than 0.01 were selected as the basis for constructing coexpression networks. Outlier samples were removed by setting a CutHeight threshold of 120. In the case of the RA dataset GSE55235 [18], genes with a MAD larger than 0.01 and in the top 75% of the MAD were applied. We used soft-thresholding powers of 5 and 9 to create CRC and RA co-expression networks, respectively, which enabled our networks to achieve a scale-free topology. Specifically, we calculated the scale independence (R^2) of the networks at different soft-thresholding powers, and selected the minimum power that resulted in an R^2 greater than 0.85, a commonly accepted criterion for scale-free networks [21]. This process was facilitated by the ‘pickSoftThreshold’ function in the WGCNA package. For visualization, hierarchical clustering dendrograms were created for both RA and CRC datasets. The criteria used were a minModuleSize of 200 and a mergeCutHeight of 0.25. By evaluating the correlations between module eigengenes (ME) and CRC, modules exhibiting a positive correlation with CRC were identified. The genes within these modules, obtained through the aforementioned steps, were considered CRC-associated genes. Similarly, we obtained a list of genes associated with RA. By intersecting the genes associated with CRC and those associated with RA, we identified a set of shared genes that are linked to both CRC and RA, which were subsequently investigated in further detail. We then conducted Gene Ontology (GO) and Kyoto Encyclopedia of Genes and Genomes (KEGG) enrichment analyses using the DAVID database. Refer to the supplementary methods for a detailed methodology.

#### Identification and prognostic value evaluation of the shared gene signature

The genes obtained from the SMR analysis were intersected with the shared genes of CRC and RA. The resulting genes were identified as shared causal genes. Nonpaired and paired tissues from the CRC cohort in the TCGA database and the RA dataset GSE55457 [18] were utilized to validate these shared causal genes. Following validation at this step, shared causal genes that still exhibit differential expression were defined as the shared gene signature between RA and CRC, which was used for subsequent analyses. We examined the relationship between the expression of the shared hub gene and patient prognosis in CRC. This involved analyzing disease-specific survival (DSS), overall survival (OS), and progression-free interval (PFI) using Kaplan‒Meier (KM) curves generated with the “survminer” and “survival” packages. The relevant data for analysis were obtained from the TCGA database. The KM grouping was determined based on the minimum *P* value grouping.

#### Validation of the expression of the shared gene signature at protein level

The Human Protein Atlas (HPA) database (http://www.proteinatlas.org/) provides valuable resources such as protein expression profiles, subcellular localization information, and immunohistochemistry images [22]. Immunohistochemical staining images of the shared hub gene were acquired from the HPA database. These images provide visual evidence showcasing the differential expression and spatial distribution of the shared hub gene in colon adenocarcinoma and normal colon tissue, as well as rectal adenocarcinoma and normal rectum tissue.

#### Immune infiltration analysis and gene‒gene interaction networks of the shared gene signature

CIBERSORT is a validated computational method enables the estimation of immune cell compositions and identification of 22 human hematopoietic cell phenotypes in various cancer types [23]. In this study, we applied the CIBERSORT algorithm to the RA dataset GSE77298 [18] and CRC dataset GSE113513 [24] to analyze the characteristics of immune cell infiltration. We utilized the GSE77298 dataset for analyzing immune infiltration differences between RA and control groups is underpinned by its demonstrated high consistency with integrated immune infiltration from three single-cell RNA sequencing datasets, as evidenced by previous research [25]. For CRC, we simultaneously employed the GSE113513 and GSE20842 datasets to investigate differences in immune infiltration between CRC and control groups. The immune infiltration results derived from these two datasets can be cross-verified, thereby fostering more reliable conclusions. Furthermore, we utilized the GeneMANIA database (http://genemania.org/) to investigate the interactions between the shared hub gene and its associated genes, providing insights into the biological mechanisms and functions in which these genes are involved.

#### Exploring potential shared mechanisms using gene set enrichment analysis (GSEA)

GSEA was conducted separately on the RA datasets (GSE55235 and GSE225731) and the CRC datasets (GSE20842, GSE113513, and GSE39582). The intersecting results from these analyses were identified as the shared pathways between RA and CRC. The samples were categorized into two groups based on the median level of gene expression. Subsequently, GSEA was conducted on these two groups of genes using GSEA software. The h.all.v2023.1.Hs.symbols.gmt gene set and c2.cp.kegg.v2023.1.Hs.symbols.gmt were downloaded as references. Filtering criteria were applied, considering normalized enrichment score (|NES|) > 1, q value < 0.25, and *P* < 0.05.

#### Single-cell transcriptome analyses

Tumor Immune Single-cell Hub 2 (TISCH2) is a comprehensive scRNA-seq database (http://tisch.comp-genomics.org/) that offers detailed cell-type annotation at the single-cell level [26]. The single-cell RNA sequencing dataset GSE146771 [27] from TISCH2 was utilized to investigate the expression of the shared gene signature at the single-cell level and to examine the enriched pathways.

#### Predictive analysis of MYO9A in CRC immunotherapy response based on mutation status

The immunotherapy response predictive analysis of MYO9A was conducted using the CAMOIP (http://www.camoip.net/) online tool [28]. We conducted a multivariable Cox regression analysis, considering OS in CRC as the outcome variable. The analysis included stage, MYO9A (mutant type vs. wild type), gender, and age variables. Based on the occurrence of MYO9A gene mutations, a gene mutation landscape between the mutant and wild-type groups was plotted. Additionally, differences in tumor mutation burden (TMB), neoantigen loads, and MANTIS score between MYO9A mutant and wild-type groups were calculated.

#### Pan-cancer analysis evaluating the expression pattens and prognostic role of MYO9A in 33 human tumors

To further investigate the potential role of MYO9A in human cancers, we performed pan-cancer analysis. We downloaded and curated RNAseq data in TPM format from 33 tumor types projects using the STAR pipeline from the TCGA database. Wilcoxon rank sum test was used to analyze the differential expression of MYO9A between tumor and normal groups in 33 tumor types. Prognostic effect analysis of MYO9A in pan-cancer was also performed using Kaplan-Meier Plotter online tools (https://kmplot.com/analysis/index.php?p=service&cancer=pancancer_rnaseq), with OS being selected as the indicator.

## Results

### Results of MR analysis and SMR analysis

The findings of the MR analysis are presented in Table S4. For discovery datasets, MR analysis showed that genetically predicted RA was associated with a slight increased risk of CRC (IVW: OR = 1.04, 95% CI = 1.01–1.07, *P* = 0.005). In sensitivity analyses, the MR‒Egger intercept test showed no evidence of unbalanced pleiotropy (P _intercept_ = 0.648). Although heterogeneity was detected by Cochran’s Q test (P _heterogeneity_ = 1.03 × 10^− 4^), consistent significant associations were demonstrated by both the random-effects IVW model (OR = 1.04, 95% CI = 1.01–1.07, *P* = 0.005) and the fixed-effect IVW model (OR = 1.04, 95% CI = 1.02–1.06, *P* = 9.58 × 10^− 7^). This suggests that there is evidence supporting the association between RA and CRC [29]. Consistent findings were replicated in the validation datasets, indicating an association between genetically predicted RA and a slight increased risk of CRC (IVW: OR = 1.04, 95% CI = 1.01–1.08, *P* = 0.035). The MR‒Egger intercept test revealed no indications of unbalanced pleiotropy (P _intercept_ = 0.322). Cochran’s Q test indicated no presence of heterogeneity. Leave-one-out test indicated no outliers (Fig. S1). The SMR analysis revealed a total of 71 genes that exhibited significant associations with CRC. These 71 genes were identified as CRC-related causal genes, with MYO9A being one of the causal genes associated with a decreased risk of CRC (OR _SMR_=0.82, P_SMR_=1.73 × 10^− 6^) (Table S5).

### Results of bioinformatic analyses

#### WGCNA identified RA-related genes and CRC-related genes

**Fig. 2:**
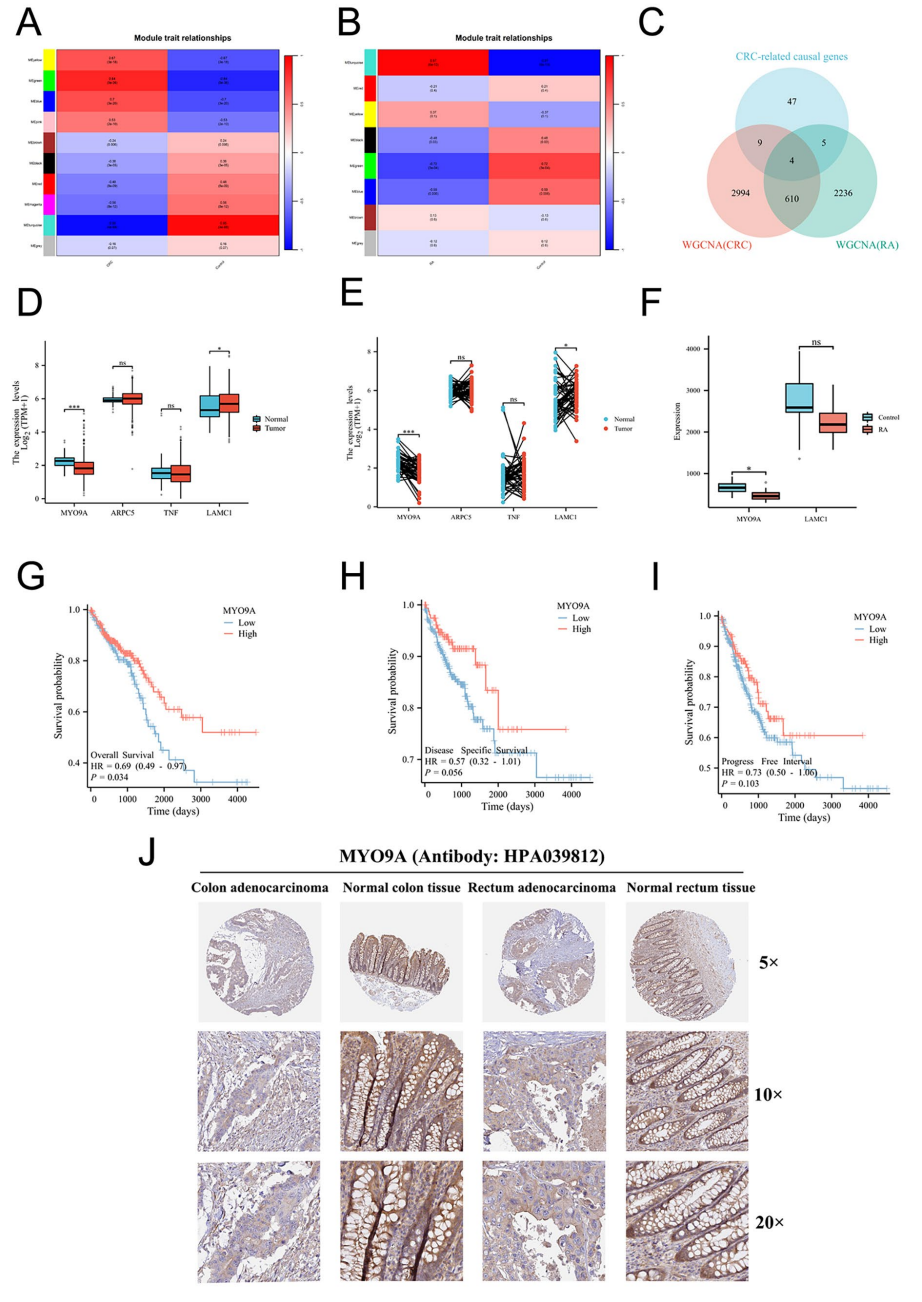
(**A**) Heatmap of module-trait relationships in CRC. (**B**) Heatmap of module-trait relationships in RA. (**C**) Intersection of WGCNA results of CRC, WGCNA results of RA and CRC-related causal genes. (**D**) Validation of four shared causal genes in nonpaired samples from TCGA-CRC cohort. (**E**) Validation of four shared causal genes in paired samples from the TCGA-CRC cohort. (**F**) Validation of four shared causal genes in RA dataset. (**G**) Overall survival. (**H**) Disease-specific survival. (**I**) Progression-free interval. (**J**) Validation of MYO9A expression at protein levels

The results of WGCNA were presented in Fig. 2A-B and Fig. S2. WGCNA was applied to the CRC dataset GSE20842, resulting in 129 remaining samples after eliminating outliers. The optimal soft threshold of 5 was determined using the pickSoftThreshold function with R^2^ = 0.85 as the scale-free topology criterion. Three modules (green, blue, and yellow) showed the strongest positive correlation with CRC. A total of 3618 CRC-related genes were identified. Similarly, in the RA dataset GSE55235, the optimal soft threshold was determined to be 9, and the turquoise module displayed the strongest association with RA. A total of 2856 RA-related genes were identified. The intersection of CRC-related genes and RA-related genes represents the shared genes between CRC and RA. GO and KEGG analyses were conducted on these shared genes, and the results are presented in Fig. S3.

#### MYO9A was identified as the shared gene signature and was associated with CRC prognostic

Four shared causal genes, namely, ARPC5, TNF, LAMC1, and MYO9A, were identified through the intersection of 45 CRC-related causal genes obtained from SMR analysis and the shared genes of CRC and RA (Fig. 2C). The validation of the shared causal genes was conducted using a cohort of CRC samples from TCGA (Fig. 2D-E) and RA datasets GSE55457 (Fig. 2F). Among the shared causal genes, MYO9A demonstrated consistent differential expression through the validation process and was identified as the shared hub gene between RA and CRC. Downregulation of MYO9A expression was observed in CRC tissues. Prognostic evaluation of MYO9A was conducted through survival analysis. The hazard ratios (HRs) for MYO9A in terms of OS, DSS, and PFI were calculated as 0.69 (*P* = 0.034), 0.57 (*P* = 0.056), and 0.73 (*P* = 0.103), respectively. These findings suggest that lower expression of MYO9A is associated with a poorer prognosis in CRC (Fig. 2G-I). We further analyzed MYO9A expression at the protein level in colon adenocarcinoma, normal colon tissue, rectal adenocarcinoma, and normal rectum tissue using data available in the HPA database. Antibody HPA 039812 was used for immunohistochemical staining of MYO9A. The results obtained from immunohistochemical staining were consistent with the transcriptional level observed before, further validating the reliability of MYO9A that we found (Fig. 2J).

#### Immune infiltration analysis revealed correlations between MYO9A expression and immune cell markers

Fig S4 show the results of immune infiltration analysis. Plasma cells were found to be downregulated in CRC but upregulated in RA. In both diseases, MYO9A expression showed a negative correlation with the enrichment of activated memory CD4 + T cells and M0 macrophages, while a positive correlation was observed with the enrichment of resting mast cells. Additionally, MYO9A expression displayed a positive correlation with plasma cells in CRC, but a negative correlation in RA.

#### Gene-gene interaction networks revealed MYO9A was associated with polarized epithelial cell differentiation

**Fig. 3:**
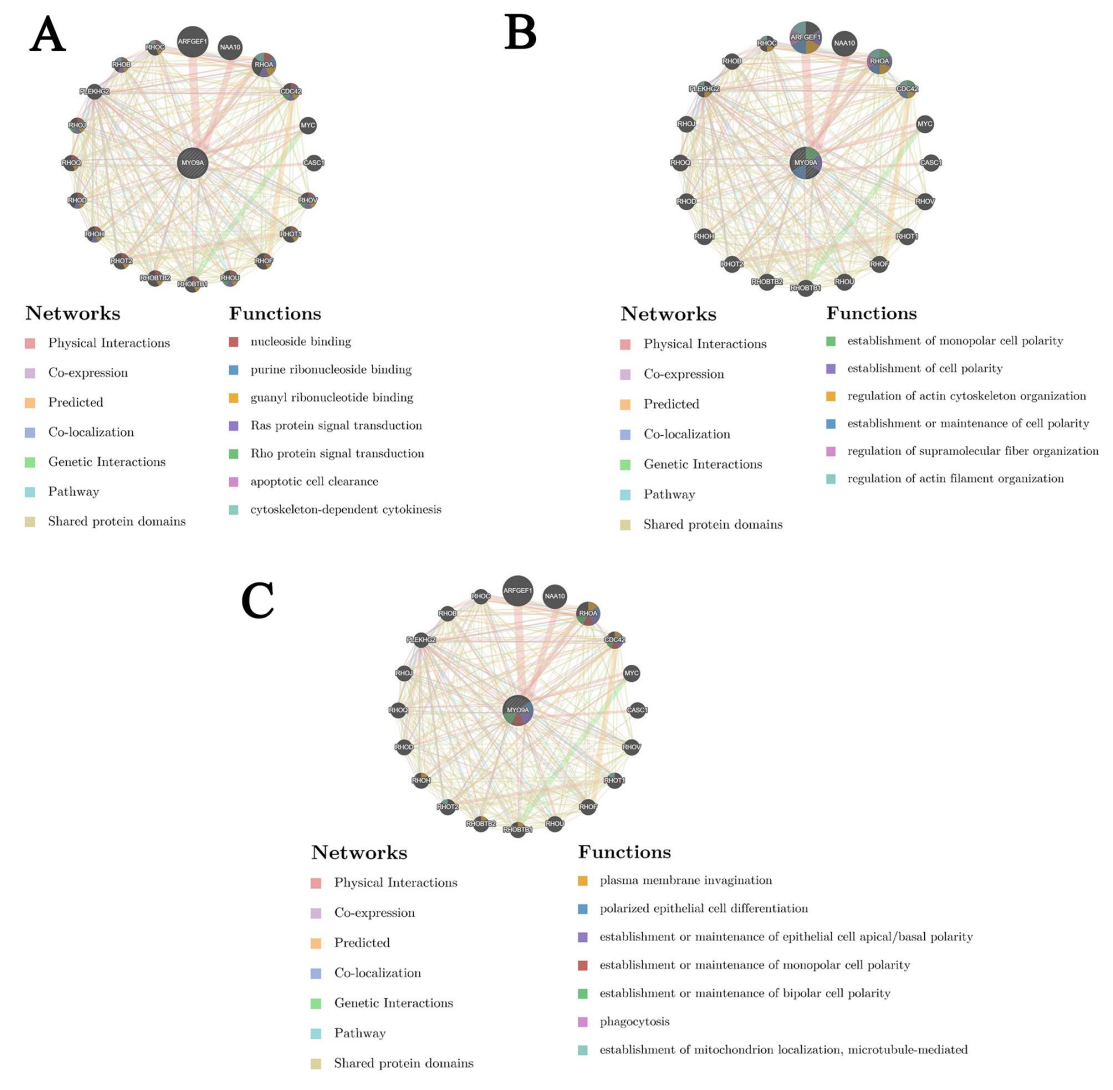
Biological processes associated with MYO9A and its related genes. (**A**) MYO9A closely interacts with the RHO family. (**B**-**C**) The main function of MYO9A is polarized epithelial cell differentiation establishment or maintenance of epithelial cell apical/basal polarity

GeneMANIA analysis illustrated that the genes that exhibit significant interactions with MYO9A (Fig. 3A-C). Interestingly, we found that MYO9A is closely associated with the Rho family in terms of biological functions. The Rho family has been proven to play a crucial role in tumorigenesis-related cytoskeletal regulation, cell polarity establishment, and cell motility [30]. In addition, our results revealed that the biological functions of MYO9A and its related genes are primarily associated with polarized epithelial cell differentiation, the establishment or maintenance of establishment of cell polarity, phagocytosis, apoptotic cell clearance, and DNA synthesis-related functions.

#### GSEA combining single-cell analyses revealed potential mechanisms contributing to the reduced risk of CRC in RA patients

**Fig. 4:**
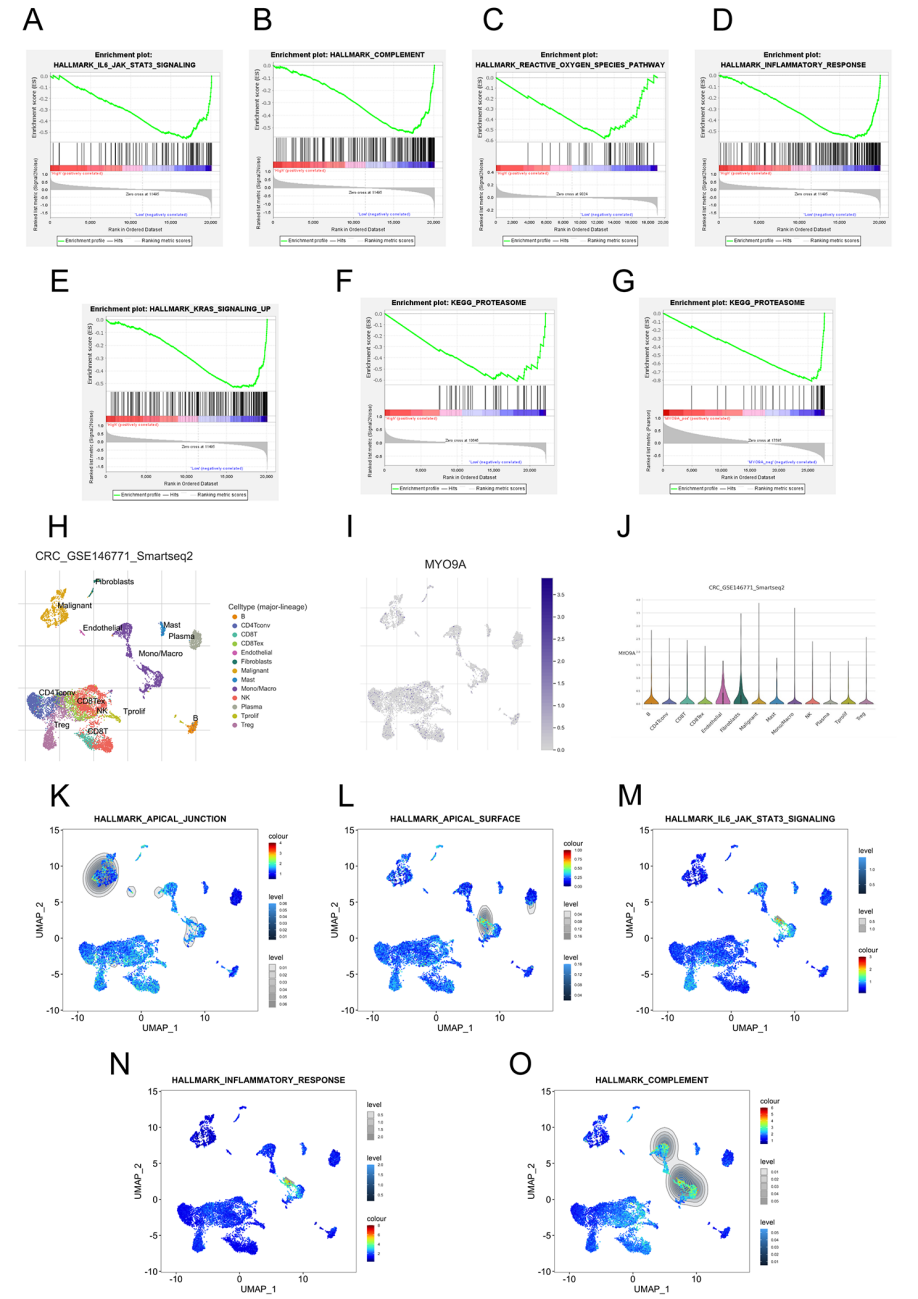
GSEA results from bulk RNA-seq and sc-RNA seq analyses. (**A**-**E**) The significantly enriched gene sets in the low MYO9A expression group in CRC. (**F**-**G**) Figures F and G respectively demonstrate significant enrichment of the Proteasome pathway in CRC and RA, indicating that this pathway is a shared pathway in both conditions. (**H**) The identified cell clusters in CRC tissues based on the GSE146771 dataset. (**I**-**J**) MYO9A had relatively high expression level in macrophages and malignant cells. (**K**-**O**) The significantly enriched gene sets in macrophages and malignant cells based on the GSE146771 scRNA-seq data

GSEA was utilized to explore the biological characteristics associated with MYO9A, and the results are presented in Tables S7-8. There was a notable enrichment of low MYO9A expression in several pathways, including reactive oxygen, IL6-JAK-STAT3, complement, KRAS signaling pathways, and inflammatory response signaling pathways (Fig. 4A-E). The proteasome pathway was identified as the shared pathway between RA and CRC, exhibiting enrichment in the low MYO9A expression group in both diseases (Fig. 4F-G). Single-cell analysis revealed a high expression of MYO9A in macrophages and malignant cells (Fig. 4H-J). Importantly, the apical junction pathway was significantly enriched in malignant cells, whereas the apical surface, complement, IL6-JAK-STATs, and inflammation response pathways were significantly enriched in macrophages (Fig. 4K-O).

#### Predictive potential of MYO9A in CRC immunotherapy response

**Fig. 5:**
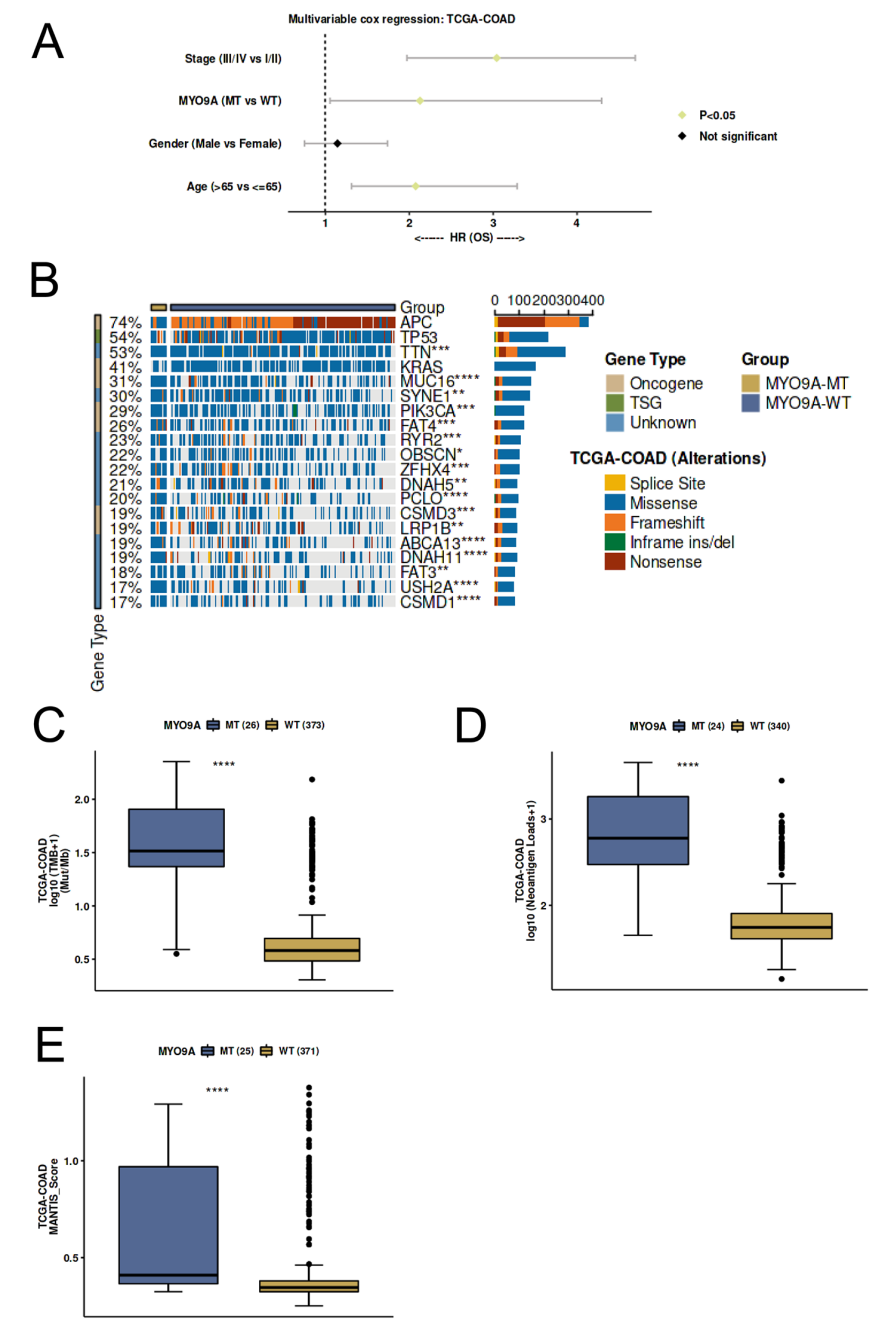
The predictive role of MYO9A in immunotherapy response. (**A**) Multivariable Cox regression analysis revealed that the mutation status of MYO9A serves as an independent predictor of overall survival. (**B**) The mutational landscape between MYO9A mutant and wild-type groups. (**C**) The association between TMB and MYO9A mutant status. (**D**) The association between neoantigen loads and MYO9A mutant status. (**E**) The association between MANTIS score and MYO9A mutant status

Multivariable cox regression analysis with OS as the outcome demonstrated a significant association between MYO9A mutation and worse OS compared to the wild-type (Fig. 5A). The mutational landscape between MYO9A mutant and wild-type groups revealed differential mutation frequencies in multiple genes, with TTN ranking first, which encodes another myofilament protein. This finding suggests a potential interaction or synergy between MYO9A and TTN, collectively involved in the development and progression of CRC (Fig. 5B). Importantly, the results of immunogenicity analysis indicated significantly higher TMB, neoantigen loads, and MANTIS score in MYO9A mutant patients compared to the wild-type (Fig. 5C-E). These results suggest that MYO9A has the potential to serve as a predictive marker for the efficiency of CRC immunotherapy.

#### Pan-cancer analyses demonstrated MYO9A plays crucial role in tumorigenesis and tumor development

**Fig. 6:**
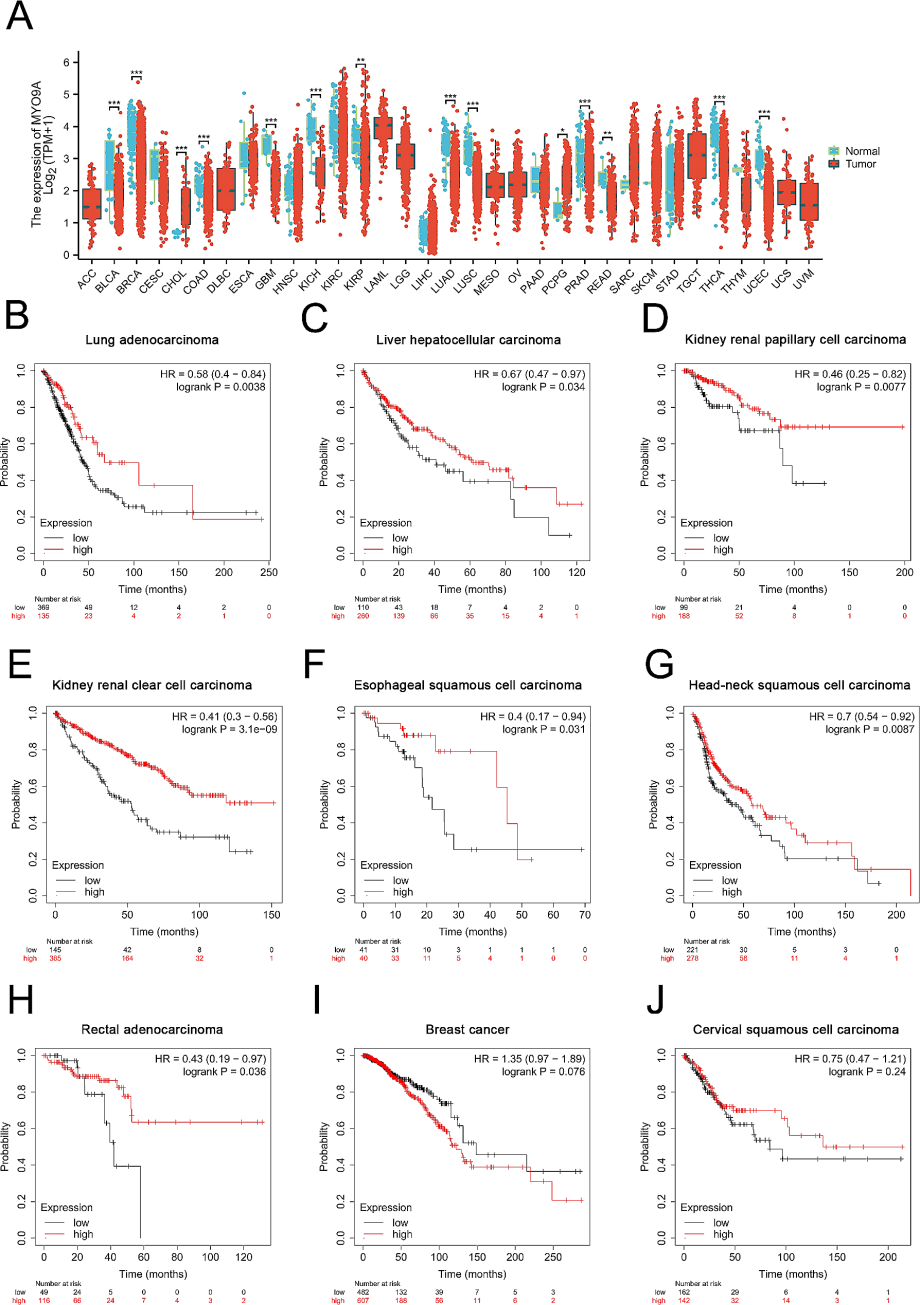
Pan-cancer analysis of MYO9A. (**A**) Expression difference of MYO9A in 33 types of tumors. (**B**-**J**) Prognostic analysis of overall survival comparing MYO9A-high and -low patients in LUAD, LIHC, KIRP, KIRC, ESCA, HNSC, READ, BRCA, and CESC. Data were analyzed using the KM plot online tool with GEO datasets

The results of expression pattern analyses showed that, except for cholangiocarcinoma and pheochromocytoma and paraganglioma, MYO9A exhibited significantly lower expression in 12 types of cancer tissues, including bladder cancer, breast cancer, renal carcinoma, and lung cancer, compared to normal tissues (Fig. 6A). Results of overall survival analysis of MYO9A in pan-cancer were presented in Table S6. Survival analyses revealed that MYO9A acted as a favorable prognostic factor in 7 types of cancer tissues, as lower expression of MYO9A was significantly associated with poorer overall survival (Fig. 6B-H). In conclusion, these findings indicate that MYO9A may play a crucial role in the process of human tumorigenesis and tumor development.

## Discussion

This study is the first to evaluate the causality and potential molecular mechanisms behind RA and CRC by multi-omics analysis. Previous research has suggested a potential correlation between immune-mediated diseases and an increased or decreased risk of cancer in distant organs or systemically, extending beyond local organ involvement [6]. RA, as a systemic immune-mediated disease, has been reported positively linked to an increased risk of lung cancer and lymphoma. Conversely, it has been observed inversely associated with the risk of prostate cancer and breast cancer [6]. Nevertheless, there is still inconclusive evidence regarding the association between RA and CRC. Our study demonstrated that RA might be associated with a slight increased risk of CRC in two independent datasets, further identifying MYO9A as a shared hub gene between RA and CRC. We observed downregulation of MYO9A in CRC, which was associated with poorer prognosis in CRC patients. Similar trend was observed in multiple solid tumors through pan-cancer analysis. Additionally, we revealed that MYO9A could serve as a robust predictive biomarker for both cancer prognosis and immunotherapy response in CRC. In addition, pan-cancer analyses demonstrated MYO9A may have a protective role in the occurrence and progression of various human cancers, such as bladder cancer and breast cancer. Previous studies have shown that myosin-9 can inhibit cervical cancer tumorigenesis by regulating autophagy [31], and participate in inhibiting breast cancer cell proliferation and invasion [32], which confirmed our findings.

Dysregulation of the actin cytoskeleton plays a pivotal role in the progression of CRC, facilitating cancer cell proliferation and metastasis. Myosin motors play a crucial role in regulating the architecture and remodeling of the actin cytoskeleton. They act as mechanosensors within the tumor microenvironment and control vital cellular processes implicated in oncogenesis. These processes include cell division, adhesion to the extracellular matrix, and tissue invasion [33]. The human genome comprises 40 genes that code for different types of myosins. These myosins can be categorized into 13 distinct classes, which are determined by their specific sequence and structural characteristics [34]. There is growing evidence indicating that certain members of the myosin superfamily, such as I, V, VI, X, and XVIII, possess either tumor-promoting or tumor-suppressing properties in CRC [33]. Nevertheless, the precise involvement of MYO9A in CRC tumorigenesis remains poorly elucidated, primarily due to the intricate nature of its functions and upstream regulatory mechanisms. Our findings revealed a significant downregulation of MYO9A expression in CRC, which was strongly correlated with poorer overall survival. SMR analysis supported that MYO9A was a causal gene associated with a decreased risk of CRC. These findings support the role of MYO9A as a tumor suppressor gene in CRC.

Gene-gene interaction analysis revealed that MYO9A and its associated genes, especially the RHO family, play a significant role in various crucial cellular processes, including the polarized epithelial cell differentiation, establishment and maintenance of epithelial cell apical/basal polarity, phagocytosis, and apoptotic cell clearance. As an unconventional myosin with a Rho-GAP domain, MYO9A regulates epithelial cell junctions, actin crosslinking, and the deactivation of RhoA, a key protein in actin stress fiber formation and contractility [30]. Loss of polarity and disruption of epithelial architecture have been observed in advanced tumor progression and metastasis, as demonstrated by previous studies [35]. Gandalovičová A et al. reported that reduced cell polarity and differentiation are associated with enhanced motility, invasion, and metastatic capabilities of epithelial cells [36]. Additionally, the disruption of cell polarity can cause the abnormal localization of degradative metalloproteinases on the cell surface, which in turn facilitates cell invasion and transformation. However, the specific contribution of these alterations during the early stages of premetastatic tumorigenesis remains unknown. Based on current evidence and our findings, we speculate that the downregulation of MYO9A may contribute to tumorigenesis by inducing the loss of polarity and epithelial architecture. Interestingly, our single-cell analysis revealed a significant enrichment of the apical junction pathway in malignant cells, which is closely associated with the maintenance of epithelial polarity. Zhu et al. found that the establishment of apical junctions is positively related to epithelial differentiation [37]. These results support our findings regarding the loss of polarity and epithelial architecture in CRC tumorigenesis, indicating a potential involvement of the apical junction pathway. Considering that most solid tumors typically originate from aberrantly proliferating epithelial cells, we also analyzed the expression pattern of MYO9A and its association with tumor prognosis in pan-cancer. Interestingly, MYO9A was found to be downregulated in the majority of solid tumors, and its low expression was significantly correlated with poorer OS. These findings suggest that MYO9A may have a protective role in the occurrence and progression of various human cancers.

Our study also revealed that MYO9A is associated with immune infiltration in CRC, potentially contributing to its impact on prognosis. Specifically, we observed a significant negative correlation between MYO9A expression levels and the infiltration of activated memory CD4 + T cells and M0 macrophages in CRC. Choi et al. reported that dysregulated activation and expansion of CD4 + memory T cells are implicated in colitis development, which subsequently contributes to the pathogenesis of colitis-associated colon cancer [38]. Zheng H et al. found that higher infiltration of M0 macrophages is associated with worse prognosis in CRC [39]. Furthermore, immunogenicity analysis revealed a significant positive association between MYO9A mutations in CRC and higher TMB, neoantigen loads, and MANTIS score, indicating that patients with MYO9A mutations are more likely to benefit from immunotherapy. These findings suggest that MYO9A could potentially serve as a predictive biomarker for the prognosis and immunotherapy response of CRC.

Another significant finding of our study is the role of MYO9A in the pathogenesis of RA. Specifically, we observed a significantly lower expression of MYO9A in RA tissue compared to normal joint synovial tissue. A prior study demonstrated a higher prevalence of autoantibodies against cytoskeletal proteins, specifically IgG autoantibodies against myosin, in the serum of rheumatoid arthritis patients compared to compared to healthy individuals as well as patients with two other autoimmune connective tissue diseases, progressive systemic sclerosis, and systemic lupus erythematosus [40]. Thus, we speculate that the downregulation of MYO9A may be linked to abnormal myosin expression, leading to cytoskeletal reorganization, altered cell motility, and perturbed myosin functionality, potentially culminating in the production of autoantibodies against myosin. Further research is required to validate and comprehensively elucidate the intricate mechanisms involved in the role of MYO9A in RA. If the aforementioned hypothesis is confirmed, MYO9A could potentially emerge as a promising therapeutic target for RA treatment. In our investigation of immune infiltration, we observed a significant inverse association between MYO9A expression and the infiltration of activated memory CD4 + T cells in patients with RA. The infiltration of CD4 + T cells, which are known as crucial effectors in the immune response, has been observed in the inflamed synovial membrane. This dysregulated immune response mediated by CD4 + T cells is widely recognized as a primary pathogenic mechanism in RA, thus corroborating our findings.

The GSEA results from bulk RNA sequencing revealed that low MYO9A expression in CRC was enriched not only in a series of common CRC-related pathways [41], such as KRAS signaling pathway and reactive oxygen pathway, but also in inflammation and immune response-related pathways, such as the IL6-JAK-STAT3 signaling pathway, complement pathway, and the inflammatory response pathway. The GSEA results from single-cell sequencing validated these findings and further indicated a significant enrichment of these pathways, mostly observed in macrophages. Talaat IM et al. demonstrated that complement activation may induce chronic inflammation, leading to the establishment of an immunosuppressive microenvironment and the activation of angiogenesis, thereby triggering signaling pathways that promote cancer development [42]. Moreover, dysregulated activation of the complement system has been established as a key contributor to the pathogenesis of diverse inflammatory and autoimmune diseases, such as RA. This is evident in two notable instances: the activation of complement on the surface of articular cartilage and within the synovium in RA, as well as complement activation induced by processes involving glycosylation, citrullination, and/or carbamylation in RA [43]. The low-MYO9A group shared an enrichment pathway—the ubiquitin‒proteasome system (UPS), which also shows great enrichment in KEGG analysis of shared genes between RA and CRC. It is a main pathway of protein degradation in cells, which maintains cell protein homeostasis. Dysfunction of the UPS plays a significant role in the onset and progression of both autoimmune diseases and malignant tumors [44]. The proteasome plays a crucial role in promoting the production of cytokines, including IL-6, and mediating the inflammatory response. Consequently, it actively contributes to the pathogenesis of RA [45]. Meanwhile, a large number of secreted cytokine networks activate STAT3 in colon epithelial cells and help induce carcinogenic transcription factors in colon cells, thereby promoting cell survival and uncontrolled proliferation and promoting the development of CRC [46]. Taken together, these findings indicate that MYO9A might serve as a shared mediator in the signaling pathways of both RA and CRC.

Our study is the first to integrate multi-omics data to investigate the association between RA and CRC, providing a comprehensive elucidation of the potential pathogenic mechanisms underpinning both diseases. However, there are some limitations to our study. Firstly, to mitigate the potential bias arising from population stratification, we employed GWAS data specifically derived from individuals of European ancestry in our MR study [47]. Thus, the findings should be generalized to other ancestries with caution. While the significance association of our MR results is supported by the currently recognized principal method, the IVW approach [29, 48, 49], and validated in another independent dataset, not all five MR methods consistently yielded significant associations. These varying OR values may reflect the different handling of statistical assumptions, confounding factors, and genetic heterogeneity by different methods [50–52]. However, this doesn’t imply contradictory evidence [53, 54]. It is important to understand that, causal inference should not rely on a single method. If a causal relationship is confirmed by multiple methods, especially when these methods make different assumptions, it becomes more robust. Moreover, although our IVW results demonstrate a significant association, the effect size of this association is not particularly strong. Therefore, further prospective studies are needed to confirm our findings. If validated, it is crucial to enhance awareness and develop personalized colorectal cancer screening strategies for individuals with rheumatoid arthritis. Finally, our results of the shared genes and pathways of RA and CRC remain at the level of data analysis. Additional molecular biology-based experiments are required to validate and corroborate our findings.

## Conclusions

MYO9A may play a role in tumor suppression, while its downregulation may contribute to the loss of polarity and epithelial architecture in tumorigenesis. Additionally, MYO9A shows promise as a powerful predictive biomarker for cancer prognosis and immunotherapy response in CRC. These findings enhance our understanding of the shared underlying biology between RA and CRC and suggest that targeting the MYO9A-mediated loss of polarity and epithelial architecture could be a novel therapeutic approach for CRC.

### Electronic supplementary material

Below is the link to the electronic supplementary material.

Supplementary Material 1

Supplementary Material 2

Supplementary Material 3

Supplementary Material 4

Supplementary Material 5

## Data Availability

The data used for MR analyses were obtained from GWAS catalog (https://www.ebi.ac.uk/gwas/) at GCST90255675 and GCST012879; IEU Open GWAS Project (https://gwas.mrcieu.ac.uk/) at ebi-a-GCST005569 and ieu-a-832. The eQTL data were obtained from the eQTLGen consortium (https://www.eqtlgen.org/phase1.html). The data used for bioinformatic analyses were obtained from Gene Expression Omnibus (GEO) at GSE55235, GSE77298, GSE55457, GSE225731, GSE20842, GSE113513 and GSE39582.

## Abbreviations

CRC: Colorectal cancer
DSS: Disease-specific survival
HEIDI: Heterogeneity in dependent instruments
HPA: Human Protein Atlas
HR: Hazard ratio
KM: Kaplan-Meier
MAD: Median absolute deviation
ME: Module eigengenes
MR: Mendelian randomization
RA: Rheumatoid arthritis
TCGA: The Cancer Genome Atlas
UPS: Ubiquitin-proteasome system
