# Supplementary material for: Integrated multi-omics analyses revealed the association between rheumatoid arthritis and colorectal cancer: MYO9A as a shared gene signature and an immune-related therapeutic target
Source: BMC Cancer. 2024 Jun 10;24:714. doi: 10.1186/s12885-024-12466-5 (PMC11165834; doi:10.1186/s12885-024-12466-5)
Supplement: Supplementary file 1 — Supplementary Material 1 [file 12885_2024_12466_MOESM1_ESM.docx]

### Supplementary Material

### Supplementary Methods

#### Detailed statistical methods for Mendelian randomization

Multiple MR approaches were employed to evaluate the causal relationship between RA and CRC. These methods included the inverse variance weighted (IVW) method, weighted mode, MR‒Egger, weighted median, and simple mode [[1](#_ENREF_1)]. Specifically, the primary MR analyses were performed by the IVW method, which provides the most precise estimates though assuming that all SNPs are valid instruments [[2](#_ENREF_2), [3](#_ENREF_3)]. The weighted median method can provide consistent estimates when more than 50% of the weight comes from valid instrument variants [[3](#_ENREF_3)]. MR-Egger regression can generate estimates after accounting for horizontal pleiotropy albeit with less precision [[3](#_ENREF_3)]. In addition, the presence of potential directional pleiotropy in the genetic variants was assessed using the MR‒Egger intercept's test. To evaluate heterogeneity, Cochran's Q test was performed [[1](#_ENREF_1)]. The leave-one-out test is utilized to identify potential outliers and assess their possible influence [[1](#_ENREF_1)]. The findings were considered statistically significant at P < 0.05.

According to Burgess et al. [[2](#_ENREF_2)], in the absence of significant heterogeneity, estimates from random and fixed effects models align without precision loss. However, in the presence of substantial heterogeneity, fixed effects may be overly precise, warranting consideration of this heterogeneity. If a causal effect is indicated by random effects analysis, it suggests evidence of a causal relationship between exposure and outcome, accounting for the observed heterogeneity. Therefore, in addressing heterogeneity, we adopt the widely accepted approach: random-effects IVW models are employed in the presence of heterogeneity, while the fixed-effect IVW model is applied in its absence [[2](#_ENREF_2), [4-6](#_ENREF_4)]. Burgess et al. have provided examples of scenarios where estimates from conventional MR methods diverge from those of other methods, discussing interpretation strategies for such cases [[7](#_ENREF_7)]. When not all of the aforementioned five MR algorithms yield significant results (P<0.05), we adopt a widely accepted criterion for determining the significance of causal associations: if the P-value for IVW is less than 0.05, the directions of IVW and MR-Egger are consistent, and no pleiotropy is detected, the result can be considered positive, regardless of whether the results from methods other than IVW are significant [[7](#_ENREF_7), [8](#_ENREF_8)].

#### Detailed statistical methods for WGCNA analysis

WGCNA is a powerful algorithm that clusters genes, constructs modules based on shared expression patterns, and explores associations with biological traits in multiple samples [[9](#_ENREF_9)]. Genes are filtered based on the median absolute deviation (MAD), a robust statistical measure often employed to assess data variability, given that genes with low expression or minimal variation typically constitute noise [[10](#_ENREF_10)]. MAD is particularly resistant to outliers, making it a suitable tool for such applications [[10](#_ENREF_10)]. In the current study, we observed substantial gene expression variability in the CRC dataset GSE20842. Consequently, we opted for a higher threshold, specifically 35% of the MAD value, to filter out noise and genes with low variability. Conversely, the RA dataset GSE55235 exhibited less gene expression variability, prompting us to select a lower threshold, namely 25% of the MAD value, to eliminate noise and genes with minimal variation. This strategy allows us to retain essential characteristics of each dataset while minimizing noise and irrelevant variation to the greatest extent possible. Specifically, in the CRC dataset GSE20842 [[11](#_ENREF_11)], genes in the top 65% MAD and MAD greater than 0.01 were selected as the basis for constructing coexpression networks. Outlier samples were removed by setting a CutHeight threshold of 120. In the case of the RA dataset GSE55235 [[12](#_ENREF_12)], genes with a MAD larger than 0.01 and in the top 75% of the MAD were applied. We used soft-thresholding powers of 5 and 9 to create CRC and RA co-expression networks, respectively, which enabled our networks to achieve a scale-free topology. Specifically, we calculated the scale independence (R^2) of the networks at different soft-thresholding powers, and selected the minimum power that resulted in an R^2 greater than 0.85, a commonly accepted criterion for scale-free networks [[10](#_ENREF_10)]. This process was facilitated by the 'pickSoftThreshold' function in the WGCNA package. For visualization, hierarchical clustering dendrograms were created for both RA and CRC datasets. The criteria used were a minModuleSize of 200 and a mergeCutHeight of 0.25. By evaluating the correlations between module eigengenes (ME) and CRC, modules exhibiting a positive correlation with CRC were identified. The genes within these modules, obtained through the aforementioned steps, were considered CRC-associated genes. Similarly, we obtained a list of genes associated with RA.

**References:**

1. Zhan ZQ, Huang ZM, Zhou HB, Xie ZX, Chen YZ, Luo YH, Chen PZ, Kang JQ, Cheng ZJ, Sun B: Gastroesophageal reflux disease with 6 neurodegenerative and psychiatric disorders: Genetic correlations, causality, and potential molecular mechanisms. J Psychiatr Res 2024, 172:244-253.

2. Burgess S, Thompson SG. Mendelian Randomization: Methods for Causal Inference Using Genetic Variants. 2nd ed. Chapman and Hall/CRC; 2021.

3. Yuan S, Chen J, Ruan X, Sun Y, Zhang K, Wang X, Li X, Gill D, Burgess S, Giovannucci E et al: Smoking, alcohol consumption, and 24 gastrointestinal diseases: Mendelian randomization analysis. Elife 2023, 12.

4. Chen Z, Chen Z, Jin X: Mendelian randomization supports causality between overweight status and accelerated aging. Aging Cell 2023, 22(8):e13899.

5. Papadimitriou N, Dimou N, Tsilidis KK, Banbury B, Martin RM, Lewis SJ, Kazmi N, Robinson TM, Albanes D, Aleksandrova K et al: Physical activity and risks of breast and colorectal cancer: a Mendelian randomisation analysis. Nat Commun 2020, 11(1):597.

6. Yuan S, Kim JH, Xu P, Wang Z: Causal association between celiac disease and inflammatory bowel disease: A two-sample bidirectional Mendelian randomization study. Front Immunol 2022, 13:1057253.

7. Burgess S, Thompson SG: Interpreting findings from Mendelian randomization using the MR-Egger method. Eur J Epidemiol 2017, 32(5):377-389.

8. Ji D, Chen WZ, Zhang L, Zhang ZH, Chen LJ: Gut microbiota, circulating cytokines and dementia: a Mendelian randomization study. J Neuroinflammation 2024, 21(1):2.

9. Stuart JM, Segal E, Koller D, Kim SK: A gene-coexpression network for global discovery of conserved genetic modules. Science 2003, 302(5643):249-255.

10. Zhang B, Horvath S: A general framework for weighted gene co-expression network analysis. Stat Appl Genet Mol Biol 2005, 4:Article17.

11. Gaedcke J, Grade M, Jung K, Camps J, Jo P, Emons G, Gehoff A, Sax U, Schirmer M, Becker H et al: Mutated KRAS results in overexpression of DUSP4, a MAP-kinase phosphatase, and SMYD3, a histone methyltransferase, in rectal carcinomas. Genes Chromosomes Cancer 2010, 49(11):1024-1034.

12. Woetzel D, Huber R, Kupfer P, Pohlers D, Pfaff M, Driesch D, Häupl T, Koczan D, Stiehl P, Guthke R et al: Identification of rheumatoid arthritis and osteoarthritis patients by transcriptome-based rule set generation. Arthritis Res Ther 2014, 16(2):R84.

### Supplementary Tables

Supplementary Table 1. Detailed information of the GWAS data used for MR analysis

| **First author** | **Datasets id** | **Reported trait** | **Case** | **Control** | **Population** |
| --- | --- | --- | --- | --- | --- |
| Huyghe JR | GCST012879 | Colorectal cancer | 19,948 | 12,124 | European |
| Fernandez-Rozadilla C | GCST90255675 | Colorectal cancer | 78,473 | 107,143 | European |
| Eyre S | ebi-a-GCST005569 | Rheumatoid arthritis | 13,838 | 33,742 | European |
| Okada Y | ieu-a-832 | Rheumatoid arthritis | 14,361 | 43,923 | European |

Supplementary Table 2. SNPs associated with potential confounders and excluded from the analysis

| SNP | Trait | Beta | SE | P | PMID* |
| --- | --- | --- | --- | --- | --- |
| Discovery datasets | | | | | |
| rs60733400 | Body mass index | 0.013 | 0.004 | 5.83E-04 | 27046222 |
| rs62401699 | Cholesterol | 0.024 | 0.007 | 3.46E-04 | 25488827 |
| rs9310852 | Waist circumference | 0.008 | 0.002 | 1.43E-04 | 29026008 |
| rs12539741 | Inflammatory bowel disease | 0.001 | 2.99E-04 | 9.80E-04 | 33930428 |
| rs9603608 | Inflammatory bowel disease | -0.071 | 0.018 | 7.17E-05 | 33930428 |
| rs62395855 | Body mass index | -0.040 | 0.007 | 7.00E-09 | 27046222 |
| Validation datasets | | | | | |
| rs72928038 | Crohn's disease | -0.034 | 0.009 | 1.60E-04 | 33930428 |
| rs2304256 | Inflammatory bowel disease | -0.039 | 0.012 | 7.31E-04 | 33930428 |
| rs34695944 | Ulcerative colitis | 0.078 | 0.016 | 1.83E-06 | 33930428 |

*References for the epidemiological evidence indicating that this SNP is associated with a potential confounder trait.

Supplementary Table 3. Detailed information of the datasets used for bioinformatic analysis

| **GSE number** | **Platform** | **Samples** | **Tissue type** | **Disease** | **Analysis** |
| --- | --- | --- | --- | --- | --- |
| GSE55235 | GPL96 | 10 RA and 10 CON | Synovial tisssue | RA | WGCNA and GSEA analysis |
| GSE77298 | GPL570 | 16 RA and 7 CON | Synovial tisssue | RA | Immunoinfiltration analysis |
| GSE55457 | GPL96 | 13 RA and 10 CON | Synovial tisssue | RA | Verification of hub genes |
| GSE225731 | GPL24676 | 74 RA | Synovial tisssue | RA | GSEA analysis |
| GSE20842 | GPL4133 | 65 CRC and 65 CON | Tumor and mucosa | CRC | WGCNA, Immunoinfiltration analysis, and GSEA analysis |
| GSE113513 | GPL15207 | 14 CRC and 14 CON | Tumor and mucosa | CRC | Immunoinfiltration analysis and GSEA analysis |
| GSE39582 | GPL570 | 443 CRC and 19 CON | Tumor and mucosa | CRC | GSEA analysis |

Supplementary Table 4. Results of MR analyses for discovery and validation datasets

| Exposure | id. exposure | Outcome | id. outcome | Method | nSNP | OR (95%CI) | P-value | P pleiotropy | P heterogeneity |
| --- | --- | --- | --- | --- | --- | --- | --- | --- | --- |
| Discovery datasets | | | | | | | | | |
| Rheumatoid arthritis | ebi-a-GCST005569 | colorectal cancer | GCST90255675 | Inverse variance weighted (multiplicative random effects) | 15 | 1.04 (1.01-1.07) | 0.005 | 0.6479724 | 1.03E-04 |
| Rheumatoid arthritis | ebi-a-GCST005569 | colorectal cancer | GCST90255675 | Inverse variance weighted (fixed effects) | 15 | 1.04 (1.02-1.06) | 9.58E-07 |  |  |
| Rheumatoid arthritis | ebi-a-GCST005569 | colorectal cancer | GCST90255675 | MR Egger | 15 | 1.03 (0.99-1.08) | 0.171 |  |  |
| Rheumatoid arthritis | ebi-a-GCST005569 | colorectal cancer | GCST90255675 | Simple mode | 15 | 0.99 (0.92-1.05) | 0.667 |  |  |
| Rheumatoid arthritis | ebi-a-GCST005569 | colorectal cancer | GCST90255675 | Weighted median | 15 | 1.03 (1.01-1.05) | 0.015 |  |  |
| Rheumatoid arthritis | ebi-a-GCST005569 | colorectal cancer | GCST90255675 | Weighted mode | 15 | 1.02 (1-1.05) | 0.092 |  |  |
| Validation datasets | | | | | | | | | |
| Rheumatoid arthritis | ieu-a-832 | colorectal cancer | GCST012879 | Inverse variance weighted | 33 | 1.04 (1.01-1.08) | 0.035 | 0.322177411 | 0.767402 |
| Rheumatoid arthritis | ieu-a-832 | colorectal cancer | GCST012879 | MR Egger | 33 | 1.06 (1.01-1.12) | 0.04 |  |  |
| Rheumatoid arthritis | ieu-a-832 | colorectal cancer | GCST012879 | Simple mode | 33 | 1.05 (0.96-1.15) | 0.294 |  |  |
| Rheumatoid arthritis | ieu-a-832 | colorectal cancer | GCST012879 | Weighted median | 33 | 1.04 (0.98-1.09) | 0.197 |  |  |
| Rheumatoid arthritis | ieu-a-832 | colorectal cancer | GCST012879 | Weighted mode | 33 | 1.04 (0.99-1.09) | 0.102 |  |  |

Supplementary Table 5. Results of SMR analysis

| Gene | SYMBOL | ProbeChr | topSNP | A1 | A2 | Freq | Beta_SMR | SE_SMR | P_SMR | P_HEIDI |
| --- | --- | --- | --- | --- | --- | --- | --- | --- | --- | --- |
| ENSG00000010327 | STAB1 | 3 | rs9855470 | A | G | 0.060636 | 0.13984 | 0.022741 | 7.78E-10 | 0.105 |
| ENSG00000026036 | RTEL1-TNFRSF6B | 20 | rs6062496 | G | A | 0.400596 | -0.742158 | 0.159388 | 3.22E-06 | 0.0423 |
| ENSG00000066933 | MYO9A | 15 | rs12901580 | A | C | 0.204771 | -0.192936 | 0.040336 | 1.73E-06 | 0.012 |
| ENSG00000073605 | GSDMB | 17 | rs12936231 | C | G | 0.485089 | 0.0387012 | 0.008782 | 1.05E-05 | 0.0159 |
| ENSG00000078747 | ITCH | 20 | rs1205344 | A | G | 0.493042 | -0.183847 | 0.042202 | 1.32E-05 | 0.14 |
| ENSG00000083838 | ZNF446 | 19 | rs3794971 | C | T | 0.219682 | -0.279992 | 0.065267 | 1.79E-05 | 0.0519 |
| ENSG00000089022 | MAPKAPK5 | 12 | rs79271898 | T | C | 0.081511 | 0.171498 | 0.036871 | 3.30E-06 | 0.116 |
| ENSG00000099326 | MZF1 | 19 | rs3794964 | C | T | 0.263419 | 0.156866 | 0.028852 | 5.42E-08 | 0.011 |
| ENSG00000099953 | MMP11 | 22 | rs11914035 | T | C | 0.101392 | -0.349532 | 0.069585 | 5.08E-07 | 0.105 |
| ENSG00000100311 | PDGFB | 22 | rs5757573 | C | T | 0.38171 | -0.100474 | 0.021834 | 4.19E-06 | 0.0261 |
| ENSG00000101210 | EEF1A2 | 20 | rs6062486 | G | A | 0.296223 | -0.542708 | 0.101731 | 9.57E-08 | 0.0462 |
| ENSG00000101464 | PIGU | 20 | rs6088552 | G | A | 0.420477 | -0.414386 | 0.093994 | 1.04E-05 | 0.114 |
| ENSG00000106077 | ABHD11 | 7 | rs13233747 | A | G | 0.355865 | 0.0787474 | 0.017635 | 7.99E-06 | 0.018 |
| ENSG00000108107 | RPL28 | 19 | rs17700376 | G | A | 0.37674 | -0.0608596 | 0.013774 | 9.94E-06 | 0.0462 |
| ENSG00000111300 | NAA25 | 12 | rs78745958 | T | C | 0.082505 | 0.214583 | 0.046147 | 3.32E-06 | 0.426 |
| ENSG00000114854 | TNNC1 | 3 | rs34332947 | T | G | 0.057654 | -0.399409 | 0.073707 | 6.00E-08 | 0.0119 |
| ENSG00000115073 | ACTR1B | 2 | rs11692435 | A | G | 0.083499 | -0.199068 | 0.029608 | 1.77E-11 | 0.198 |
| ENSG00000116171 | SCP2 | 1 | rs1242331 | A | G | 0.347913 | -0.0492476 | 0.010733 | 4.47E-06 | 0.301 |
| ENSG00000121310 | ECHDC2 | 1 | rs1242331 | A | G | 0.347913 | -0.0632144 | 0.01379 | 4.56E-06 | 0.511 |
| ENSG00000122870 | BICC1 | 10 | rs10740734 | G | A | 0.486083 | 0.221104 | 0.048557 | 5.27E-06 | 0.0354 |
| ENSG00000123810 | B9D2 | 19 | rs4803457 | T | C | 0.38171 | -0.376177 | 0.059501 | 2.58E-10 | 0.0505 |
| ENSG00000124762 | CDKN1A | 6 | rs12199346 | A | C | 0.208748 | -0.288479 | 0.04276 | 1.51E-11 | 0.0337 |
| ENSG00000125686 | MED1 | 17 | rs12450559 | G | A | 0.250497 | 0.239263 | 0.052408 | 4.99E-06 | 0.0714 |
| ENSG00000125845 | BMP2 | 20 | rs6140415 | A | G | 0.154076 | 0.324046 | 0.041498 | 5.77E-15 | 0.0412 |
| ENSG00000127837 | AAMP | 2 | rs13003334 | T | A | 0.412525 | 0.644892 | 0.098861 | 6.88E-11 | 0.0531 |
| ENSG00000130724 | CHMP2A | 19 | rs3794964 | C | T | 0.263419 | 0.593162 | 0.127682 | 3.39E-06 | 0.553 |
| ENSG00000130725 | UBE2M | 19 | rs3794964 | C | T | 0.263419 | 0.304709 | 0.062902 | 1.27E-06 | 0.0858 |
| ENSG00000131969 | ABHD12B | 14 | rs17123107 | G | A | 0.203777 | -0.113052 | 0.02424 | 3.10E-06 | 0.343 |
| ENSG00000133030 | MPRIP | 17 | rs11867934 | T | C | 0.191849 | 0.358146 | 0.084972 | 2.50E-05 | 0.626 |
| ENSG00000135862 | LAMC1 | 1 | rs11588675 | T | C | 0.426441 | 0.134403 | 0.01334 | 7.14E-24 | 0.381 |
| ENSG00000136280 | CCM2 | 7 | rs10951794 | A | G | 0.207753 | -0.0845615 | 0.015138 | 2.32E-08 | 0.0272 |
| ENSG00000137834 | SMAD6 | 15 | rs76912608 | T | C | 0.250497 | 0.512029 | 0.091023 | 1.85E-08 | 0.103 |
| ENSG00000140307 | GTF2A2 | 15 | rs6151590 | A | G | 0.355865 | 0.103072 | 0.022612 | 5.16E-06 | 0.0616 |
| ENSG00000142039 | CCDC97 | 19 | rs2241715 | A | C | 0.314115 | -0.682785 | 0.128381 | 1.05E-07 | 0.165 |
| ENSG00000143344 | RGL1 | 1 | rs1184639 | C | G | 0.365805 | -0.145762 | 0.029376 | 6.98E-07 | 0.046 |
| ENSG00000146833 | TRIM4 | 7 | rs2572010 | C | T | 0.468191 | -0.0560289 | 0.010736 | 1.80E-07 | 0.078 |
| ENSG00000161395 | PGAP3 | 17 | rs2952152 | T | C | 0.318091 | 0.110543 | 0.022272 | 6.93E-07 | 0.0855 |
| ENSG00000161405 | IKZF3 | 17 | rs907091 | T | C | 0.489066 | -0.09225 | 0.020548 | 7.14E-06 | 0.478 |
| ENSG00000162704 | ARPC5 | 1 | rs2767304 | G | A | 0.400596 | -0.075644 | 0.013681 | 3.22E-08 | 0.547 |
| ENSG00000165171 | METTL27 | 7 | rs8629 | T | C | 0.274354 | -0.0480776 | 0.008883 | 6.22E-08 | 0.259 |
| ENSG00000168769 | TET2 | 4 | rs11729069 | G | C | 0.163022 | -0.686382 | 0.158771 | 1.54E-05 | 0.021 |
| ENSG00000174851 | YIF1A | 11 | rs2155030 | G | A | 0.173956 | -0.21946 | 0.051741 | 2.22E-05 | 0.0447 |
| ENSG00000175711 | B3GNTL1 | 17 | rs9890743 | T | C | 0.296223 | -0.101005 | 0.018528 | 4.99E-08 | 0.0349 |
| ENSG00000177150 | FAM210A | 18 | rs77564907 | A | G | 0.15507 | 0.271818 | 0.06406 | 2.20E-05 | 0.738 |
| ENSG00000179295 | PTPN11 | 12 | rs12425405 | C | G | 0.082505 | 0.254444 | 0.05863 | 1.43E-05 | 0.0576 |
| ENSG00000179921 | GPBAR1 | 2 | rs11677953 | A | G | 0.366799 | -0.251128 | 0.03275 | 1.75E-14 | 0.0174 |
| ENSG00000183520 | UTP11 | 1 | rs4360494 | G | C | 0.471173 | 0.235382 | 0.031913 | 1.63E-13 | 0.108 |
| ENSG00000189143 | CLDN4 | 7 | rs1989670 | T | A | 0.274354 | -0.330597 | 0.070076 | 2.39E-06 | 0.181 |
| ENSG00000197093 | GAL3ST4 | 7 | rs11764176 | T | G | 0.26839 | -0.144762 | 0.0343 | 2.44E-05 | 0.835 |
| ENSG00000198270 | TMEM116 | 12 | rs4767068 | G | A | 0.167992 | 0.0938929 | 0.02024 | 3.50E-06 | 0.576 |
| ENSG00000198324 | PHETA1 | 12 | rs11065884 | G | A | 0.224652 | 0.276074 | 0.061125 | 6.29E-06 | 0.823 |
| ENSG00000203999 | LINC01270 | 20 | rs1971480 | G | T | 0.311133 | 0.14137 | 0.029135 | 1.22E-06 | 0.0106 |
| ENSG00000204147 | ASAH2B | 10 | rs2820760 | A | G | 0.162028 | -0.681977 | 0.158217 | 1.63E-05 | 0.32 |
| ENSG00000204644 | ZFP57 | 6 | rs416568 | A | T | 0.246521 | 0.0410055 | 0.008607 | 1.89E-06 | 0.0698 |
| ENSG00000213533 | STIMATE | 3 | rs1986656 | T | C | 0.117296 | -0.18383 | 0.030666 | 2.04E-09 | 0.0457 |
| ENSG00000224397 | PELATON | 20 | rs3761181 | A | G | 0.459245 | -0.0733298 | 0.012975 | 1.59E-08 | 0.692 |
| ENSG00000226469 | ADAM1B | 12 | rs7134084 | A | G | 0.16501 | 0.113603 | 0.024782 | 4.56E-06 | 0.129 |
| ENSG00000226979 | LTA | 6 | rs2071590 | A | G | 0.363817 | -0.267616 | 0.046571 | 9.12E-09 | 0.0374 |
| ENSG00000228789 | HCG22 | 6 | rs1265054 | C | T | 0.514911 | -0.0689083 | 0.016316 | 2.41E-05 | 0.014 |
| ENSG00000229186 |  | 12 | rs12423572 | C | A | 0.082505 | -0.0684236 | 0.014561 | 2.61E-06 | 0.844 |
| ENSG00000230795 | HLA-K | 6 | rs416568 | A | T | 0.246521 | -0.0460454 | 0.009699 | 2.06E-06 | 0.133 |
| ENSG00000232810 | TNF | 6 | rs1121800 | A | T | 0.404573 | -0.166747 | 0.026172 | 1.87E-10 | 0.166 |
| ENSG00000233077 | LINC01271 | 20 | rs4811018 | A | G | 0.312127 | 0.21773 | 0.048143 | 6.11E-06 | 0.0454 |
| ENSG00000234608 | MAPKAPK5-AS1 | 12 | rs16941759 | A | G | 0.16501 | 0.104945 | 0.021916 | 1.68E-06 | 0.0841 |
| ENSG00000239732 | TLR9 | 3 | rs13098856 | A | G | 0.060636 | 0.871861 | 0.19794 | 1.06E-05 | 0.125 |
| ENSG00000258398 |  | 14 | rs11157782 | T | C | 0.202783 | -0.057633 | 0.01224 | 2.50E-06 | 0.791 |
| ENSG00000260997 |  | 7 | rs6963832 | T | C | 0.095427 | -0.0507809 | 0.00962 | 1.30E-07 | 0.0332 |
| ENSG00000261338 |  | 2 | rs736730 | C | T | 0.412525 | 0.0604115 | 0.007672 | 3.43E-15 | 0.0435 |
| ENSG00000261716 | H2BC20P | 1 | rs7531664 | C | T | 0.134195 | -0.112835 | 0.026849 | 2.64E-05 | 0.327 |
| ENSG00000262820 |  | 16 | rs11075687 | T | C | 0.214712 | 0.191731 | 0.043824 | 1.21E-05 | 0.121 |
| ENSG00000269202 |  | 20 | rs633198 | C | T | 0.483101 | 0.057938 | 0.013268 | 1.26E-05 | 0.0294 |

Supplementary Table 6. Results of overall survival analysis of MYO9A in pan-cancer.

| **Tumor type** | **Group1** | **Group2** | **Statistic** | **Difference (Tumor-Normal)** | **Confidence interval (95% CI)** | **P-value** |
| --- | --- | --- | --- | --- | --- | --- |
| BLCA | Normal | Tumor | 5717 | -0.77672 | -0.8282 | 0.0004 |
| BRCA | Normal | Tumor | 9.87E+04 | -0.8412 | -0.28113 | 2.01E-25 |
| CESC | Normal | Tumor | 657 | -0.82673 | -2.05775 | 0.19 |
| CHOL | Normal | Tumor | 37 | 0.82574 | 0.33595 - 1.3172 | 0.0002 |
| COAD | Normal | Tumor | 1.38E+04 | -0.39609 | -0.29645 | 4.82E-07 |
| ESCA | Normal | Tumor | 983 | -0.1088 | -0.95317 | 0.5692 |
| GBM | Normal | Tumor | 767 | -1.1541 | -0.92292 | 0.0006 |
| HNSC | Normal | Tumor | 9629.5 | 0.126 | -0.345524 | 0.1588 |
| KICH | Normal | Tumor | 1461 | -1.2571 | -0.65563 | 5.30E-09 |
| KIRC | Normal | Tumor | 2.17E+04 | -0.19039 | -0.422871 | 0.0697 |
| KIRP | Normal | Tumor | 6213 | -0.5131 | -0.62327 | 0.0017 |
| LIHC | Normal | Tumor | 8958.5 | 0.024041 | -0.230865 | 0.6956 |
| LUAD | Normal | Tumor | 2.56E+04 | -0.86991 | -0.32262 | 7.67E-18 |
| LUSC | Normal | Tumor | 2.29E+04 | -0.98871 | -0.29776 | 1.28E-23 |
| PAAD | Normal | Tumor | 433 | -0.2372 | -1.3015 | 0.4629 |
| PCPG | Normal | Tumor | 81 | 0.93984 | 0.083861 - 1.6187 | 0.0388 |
| PRAD | Normal | Tumor | 1.71E+04 | -0.4299 | -0.43764 | 0.0002 |
| READ | Normal | Tumor | 1308 | -0.59685 | -0.74172 | 0.0023 |
| STAD | Normal | Tumor | 5216 | 0.27492 | -0.80367 | 0.22 |
| THCA | Normal | Tumor | 2.46E+04 | -0.84152 | -0.3348 | 1.56E-16 |
| UCEC | Normal | Tumor | 1.80E+04 | -1.1967 | -0.39153 | 9.17E-19 |

*Datasets Source: Kaplan-MeierPlotter (https://kmplot.com/analysis/index.php?p=service&cancer=pancancer_rnaseq). BLCA: Bladder Cancer BRCA: Breast Cancer CESC: Cervical Cancer CHOL: Cholangiocarcinoma COAD: Colon Adenocarcinoma ESCA: Esophageal Cancer GBM: Glioblastoma HNSC: Head and Neck Squamous Cell Carcinoma KICH: Kidney Chromophobe KIRC: Kidney Renal Clear Cell Carcinoma KIRP: Kidney Renal Papillary Cell Carcinoma LIHC: Liver Hepatocellular Carcinoma LUAD: Lung Adenocarcinoma LUSC: Lung Squamous Cell Carcinoma PAAD: Pancreatic Adenocarcinoma PCPG: Pheochromocytoma and Paraganglioma PRAD: Prostate Adenocarcinoma READ: Rectal Adenocarcinoma STAD: Stomach Adenocarcinoma THCA: Thyroid Carcinoma UCEC: Uterine Corpus Endometrial Carcinoma

Supplementary Table 7. Results of GSEA analysis exploring the shared pathways involving MYO9A in CRC

| **Database** | **Description** | **ES** | **NES** | **NOM p-val** | **FDR q-val** | **FWER p-val** |
| --- | --- | --- | --- | --- | --- | --- |
| **MYO9A-HIGH EXPRESSION GROUP- CRC- GSE20842** | | | | | | |
| HALLMARK | WNT BETA CATENIN SIGNALING | 0.650 | 1.800 | 0.000 | 0.061 | 0.053 |
| HALLMARK | HEDGEHOG SIGNALING | 0.608 | 1.677 | 0.002 | 0.097 | 0.163 |
| HALLMARK | UV RESPONSE DN | 0.530 | 1.588 | 0.033 | 0.136 | 0.292 |
| HALLMARK | EPITHELIAL MESENCHYMAL TRANSITION | 0.752 | 1.578 | 0.014 | 0.110 | 0.312 |
| HALLMARK | COAGULATION | 0.514 | 1.474 | 0.021 | 0.186 | 0.517 |
| **MYO9A-LOW EXPRESSION GROUP- CRC- GSE20842** | | | | | | |
| HALLMARK | REACTIVE OXYGEN SPECIES PATHWAY | -0.586 | -2.007 | 0.000 | 0.017 | 0.009 |
| HALLMARK | OXIDATIVE PHOSPHORYLATION | -0.405 | -1.660 | 0.040 | 0.195 | 0.175 |
| **MYO9A-LOW EXPRESSION GROUP- CRC- GSE113513** | | | | | | |
| HALLMARK | ALLOGRAFT REJECTION | -0.664 | -1.815 | 0.000 | 0.022 | 0.018 |
| HALLMARK | INFLAMMATORY RESPONSE | -0.566 | -1.728 | 0.008 | 0.045 | 0.069 |
| HALLMARK | IL6 JAK STAT3 SIGNALING | -0.558 | -1.700 | 0.004 | 0.043 | 0.093 |
| HALLMARK | COMPLEMENT | -0.545 | -1.667 | 0.004 | 0.048 | 0.120 |
| HALLMARK | KRAS SIGNALING UP | -0.530 | -1.558 | 0.014 | 0.131 | 0.334 |
| HALLMARK | IL2 STAT5 SIGNALING | -0.425 | -1.426 | 0.021 | 0.234 | 0.575 |
| **MYO9A-LOW EXPRESSION GROUP- CRC- GSE39582** | | | | | | |
| KEGG | PROTEASOME | -0.611 | -1.711 | 0.000 | 0.172 | 0.122 |

Supplementary Table 8. Results of GSEA analysis exploring the shared pathways involving MYO9A in RA

| **Database** | **Description** | **ES** | **NES** | **NOM p-val** | **FDR q-val** | **FWER p-val** |
| --- | --- | --- | --- | --- | --- | --- |
| **MYO9A-HIGH EXPRESSION GROUP- RA- GSE55235** | | | | | | |
| HALLMARK | UV RESPONSE DN | 0.451 | 1.571 | 0.057 | 0.214 | 0.330 |
| **MYO9A-Low EXPRESSION GROUP- RA- GSE55235** | | | | | | |
| HALLMARK | MYC TARGETS V2 | -0.614 | -1.758 | 0.008 | 0.113 | 0.063 |
| **MYO9A-HIGH EXPRESSION GROUP- RA- GSE225731** | | | | | | |
| KEGG | PROTEASOME | -0.812 | -1.950 | 0.000 | 0.036 | 0.037 |
| KEGG | OXIDATIVE PHOSPHORYLATION | -0.686 | -1.772 | 0.016 | 0.170 | 0.257 |
