# Supplementary figures and images for: Integrated multi-omics analyses revealed the association between rheumatoid arthritis and colorectal cancer: MYO9A as a shared gene signature and an immune-related therapeutic target
Source: BMC Cancer. 2024 Jun 10;24:714. doi: 10.1186/s12885-024-12466-5 (PMC11165834; doi:10.1186/s12885-024-12466-5)

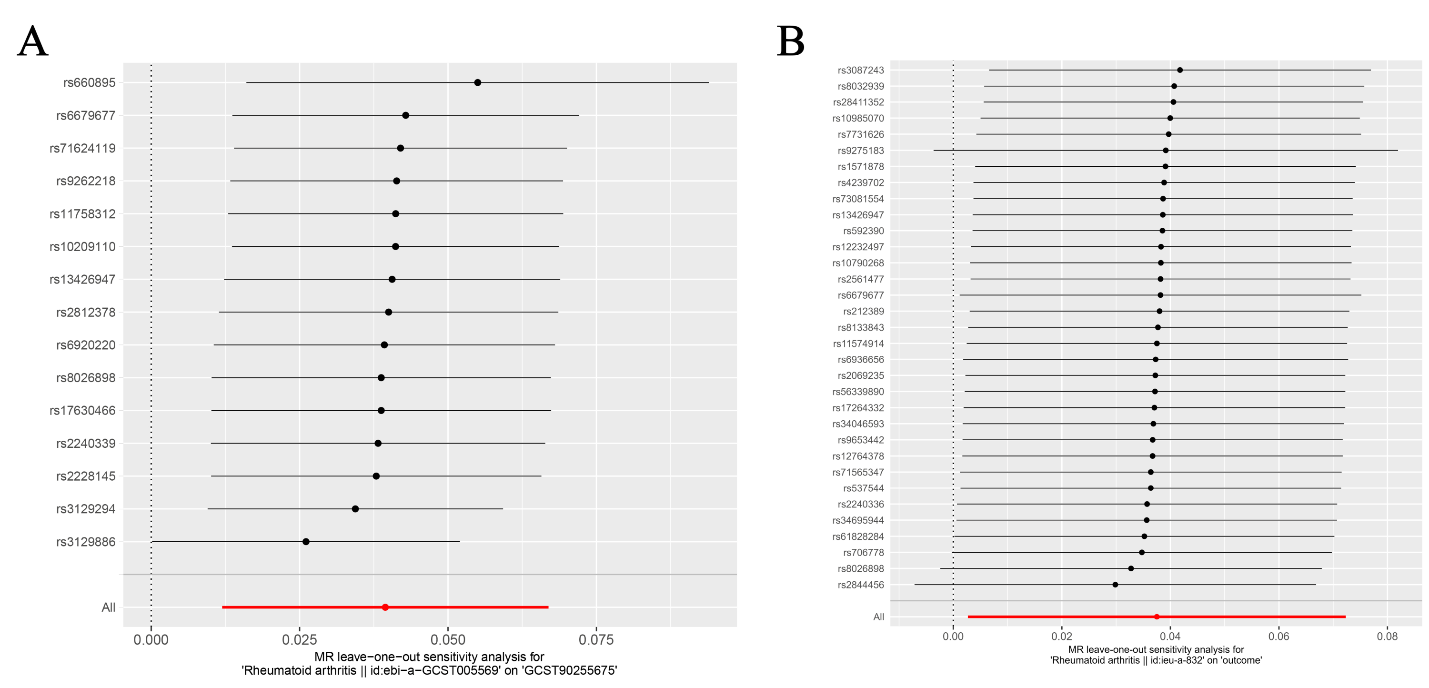


**Supplementary Fig. 1** Leave-one-out analysis for (A) discovery datasets and (B) validation

Supplement: Supplementary file 2 — Supplementary Material 2 [file 12885_2024_12466_MOESM2_ESM.docx]
