# Supplementary material for: Integrated multi-omics analyses revealed the association between rheumatoid arthritis and colorectal cancer: MYO9A as a shared gene signature and an immune-related therapeutic target
Source: BMC Cancer. 2024 Jun 10;24:714. doi: 10.1186/s12885-024-12466-5 (PMC11165834; doi:10.1186/s12885-024-12466-5)
Supplement: Supplementary file 3 — Supplementary Material 3 [file 12885_2024_12466_MOESM3_ESM.docx]

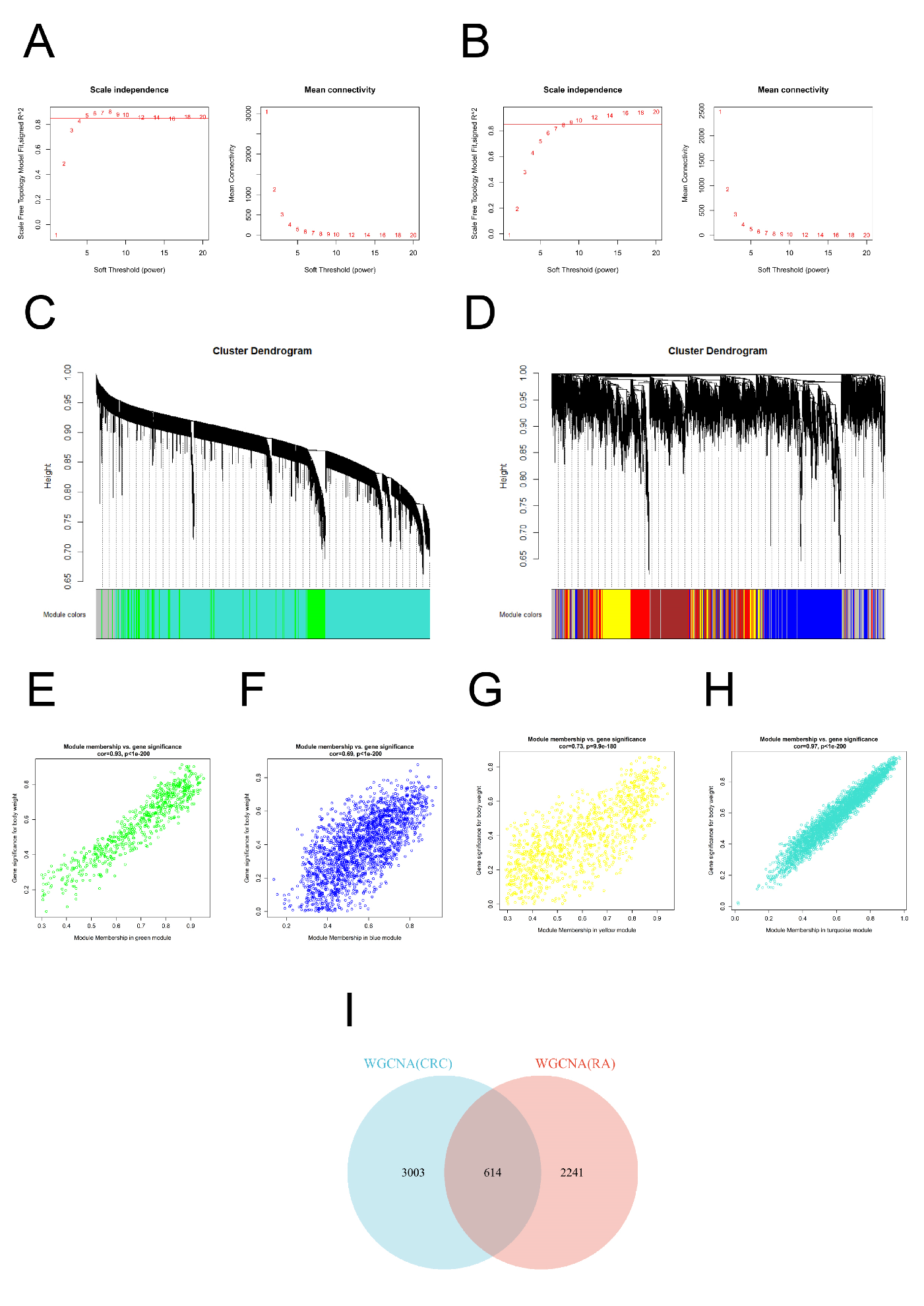


**Supplementary Fig. 2** WGCNA co-expression module construction. (A) The optimal soft-threshold power in CRC. (B) The optimal soft-threshold power in RA. (C) Cluster dendrogram of coexpressed genes in CRC. (D) Cluster dendrogram of coexpressed genes in RA. (E) Scatter plot of gene significance vs. module membership in the green module of CRC. (F) Scatter plot of gene significance vs. module membership in the blue module of CRC. (G) Scatter plot of gene significance vs. module membership in the yellow module of CRC. (H) Scatter plot of gene significance vs. module membership in the turquoise module of RA. (I) Intersection of WGCNA results of CRC and WGCNA results of RA.
