# Supplementary material for: Integrated multi-omics analyses revealed the association between rheumatoid arthritis and colorectal cancer: MYO9A as a shared gene signature and an immune-related therapeutic target
Source: BMC Cancer. 2024 Jun 10;24:714. doi: 10.1186/s12885-024-12466-5 (PMC11165834; doi:10.1186/s12885-024-12466-5)
Supplement: Supplementary file 4 — Supplementary Material 4 [file 12885_2024_12466_MOESM4_ESM.docx]

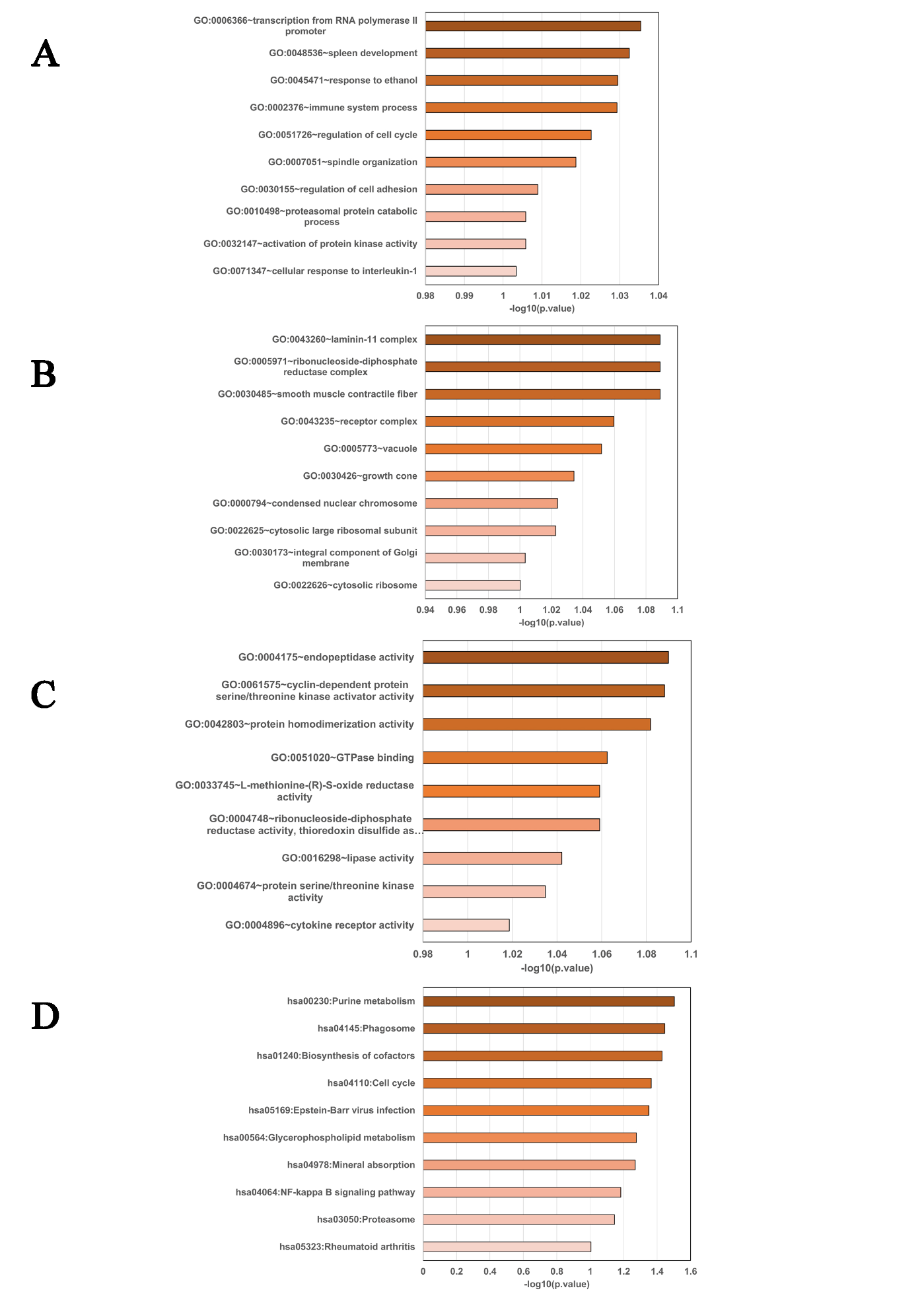


**Supplementary Fig. 3** Enrichment analysis results of shared genes in RA and CRC based on GO and KEGG enrichment analysis. (A) GO enrichment of shared genes in biological process terms. (B) GO enrichment of shared genes in cellular component terms. (C) GO enrichment of shared genes in molecular function terms. (D) Enriched KEGG pathways of the shared genes.
