# Supplementary material for: Integrated multi-omics analyses revealed the association between rheumatoid arthritis and colorectal cancer: MYO9A as a shared gene signature and an immune-related therapeutic target
Source: BMC Cancer. 2024 Jun 10;24:714. doi: 10.1186/s12885-024-12466-5 (PMC11165834; doi:10.1186/s12885-024-12466-5)
Supplement: Supplementary file 5 — Supplementary Material 5 [file 12885_2024_12466_MOESM5_ESM.docx]

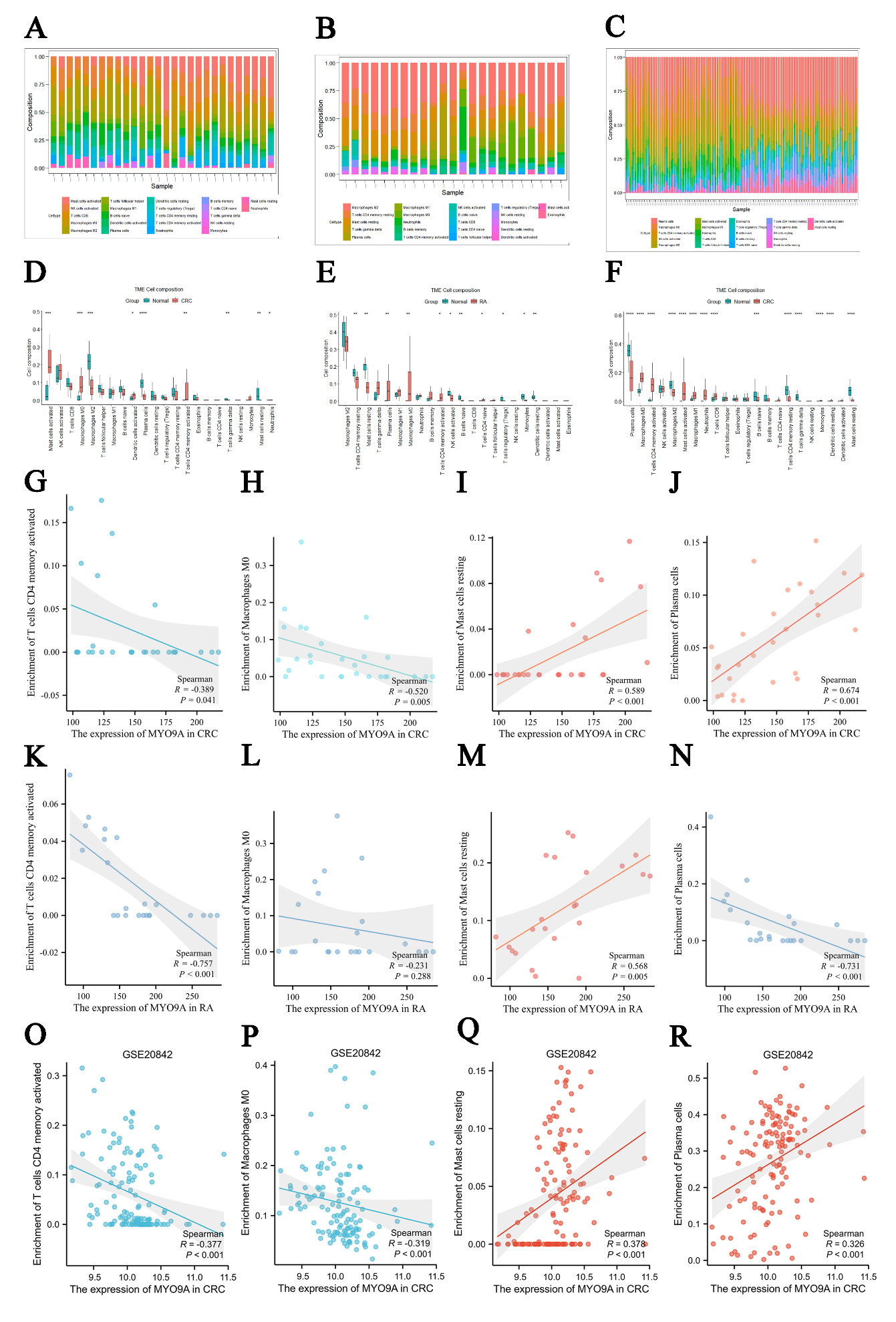


**Supplementary Fig. 4** Visualization of immune cell infiltration.

(A) Infiltration of 22 types of immune cells in individual samples in the CRC dataset GSE113513. (B) Infiltration of 22 types of immune cells in individual samples in the RA dataset GSE77298. (C) Infiltration of 22 types of immune cells in individual samples in the CRC dataset GSE20842. (D) The difference in immune infiltration proportions between the CRC and control groups (GSE113513). (E) The difference in immune infiltration proportions between the RA and control groups. (F) The difference in immune infiltration proportions between the CRC and control groups (GSE20842). (G-J) Correlation between MYO9A and activated memory T cells, M0 macrophages, resting mast cells and plasma cells in CRC (GSE113513). (K-N) Correlation between MYO9A and activated memory T cells, M0 macrophages, resting mast cells and plasma cells in RA. (O-R) Correlation between MYO9A and activated memory T cells, M0 macrophages, resting mast cells and plasma cells in CRC (GSE20842).
